# Innate immune training in the neonatal response to sepsis

**DOI:** 10.1186/s10020-025-01179-5

**Published:** 2025-04-30

**Authors:** Jaimar C. Rincon, Dayuan Wang, Valerie E. Polcz, Evan L. Barrios, Marvin L. Dirain, Ricardo F. Ungaro, Dina C. Nacionales, Leilani Zeumer-Spataro, Feifei Xiao, Philip A. Efron, Lyle L. Moldawer, Guoshuai Cai, Shawn D. Larson

**Affiliations:** 1 Sepsis and Critical Illness Research Center, Department of Surgery, University of Florida College of Medicine, 1600 SW Archer Road, P.O. Box 100119, Gainesville, FL 32610 - 0019 USA; 2 Division of Pediatric Surgery, Department of Surgery, University of Florida College of Medicine, Gainesville, FL USA; 3 Department of Biostatistics, University of Florida Colleges of Medicine and Public Health and Health Sciences, Gainesville, FL USA

**Keywords:** Neonatal sepsis, Myeloid-derived suppressor cells, BCG, Trained immunity, Single-cell RNA sequencing

## Abstract

Neonates, especially those born prematurely, are highly vulnerable to infection-induced mortality. Numerous observational and immunological studies in newborns have shown that live attenuated vaccines have beneficial, non-specific effects (NSEs) against secondary infections to unrelated pathogens. These beneficial effects have been attributed to *trained immunity*, and emergency granulopoiesis plays an essential role. However, trained immunity has been shown to affect multiple myeloid subsets and how trained immunity influences the host protective response is still undefined. Here we show that Bacillus-Calmette-Guérin (BCG) vaccination improves survival to polymicrobial sepsis by simultaneously reprogramming broad aspects of myelopoiesis. Specifically, BCG vaccination expands multiple myeloid subsets, including the lineage (Lin)^−^Sca- 1^+^c-kit^+^ (LSK) and granulocytic-macrophage progenitors (GMPs), and increases CD11b^+^Gr1^+^ cell number, as well as their oxidative metabolism and capacity to stimulate T-cell proliferation in response to sepsis. Single-cell RNA sequencing of neonatal splenocytes suggests that BCG-vaccination changes the broad transcriptional landscape of multiple myeloid subsets. The result is the maturation of various neutrophil and monocyte subsets, stimulation of antimicrobial processes, and suppression of inflammatory pathways and myeloid-derived suppressor cell transcription. These findings reveal that BCG administration early after birth fundamentally reorganizes the myeloid landscape to benefit the subsequent response to polymicrobial infection.

**Supplementary Information:**

The online version contains supplementary material available at 10.1186/s10020-025-01179-5.

## Introduction

Sepsis is a complex clinical syndrome characterized by a dysregulated inflammatory response to infection that leads to organ dysfunction and increased mortality (Singer et al. 2016). Sepsis is unfortunately common and affects all ages, with approximately 50 million cases per year worldwide and an estimated mortality of approximately 20% (Rudd et al. 2020). Newborns, especially very-low birth weight (VLBW) neonates, are highly vulnerable to infections and sepsis. In the U.S., neonatal sepsis occurs in 1–4 cases per 1000 live births, and mortality rates are inversely proportional to gestational age, ranging from 5 to 10% in VLBW neonates (Fleischmann-Struzek et al. 2018). Successful outcomes require early treatment with antibiotics and supportive care. Survivors of neonatal sepsis are at increased risk for adverse long-term developmental outcomes (Kurul et al. 2023).

Newborns are more vulnerable to severe infections than adults due to structural and functional differences in their immune system. Premature infants are heavily reliant on their innate immunity for protection, characterized by cell-intrinsic hyporesponsiveness concomitant with a T_H2_-biased response (Polcz et al. 2023) In addition, altered regulatory T cells (Tregs), myeloid-derived suppressor cells (MDSCs), and anti-inflammatory cytokine responses reduce host protective immunity in neonates, leading to an increased propensity for both sepsis and necrotizing enterocolitis (NEC) (Polcz et al. 2023; Pang et al. 2018).

Traditionally, immune memory is regarded as an exclusive hallmark of adaptive immunity; however, growing evidence suggests that early life infections can substantially shape both developing adaptive and innate immunity in neonates (Henneke et al. 2021). Trained immunity is an adaptation of innate immunity that allows the host to mount a more rapid and augmented response secondary to non-specific infectious challenges. Mediated by epigenetic and metabolic reprogramming of transcriptional pathways in response to non-specific activation, trained immunity is seen in broad classes of innate immune effector cells (Netea et al. 2016).

Live, attenuated vaccines are often protective against unrelated pathogens via non-specific effects (NSEs) (Benn et al. 2013). Epidemiological data have shown that the Bacillus Calmette-Guerin (BCG) vaccine administration early in life, can reduce all-cause neonatal mortality and lower the risk of nontargeted infectious diseases beyond the first months of life (Jensen et al. 2015; Freyne et al. 2018; Biering-Sørensen et al. 2017; Schaltz-Buchholzer et al. 2019; Bardenheier et al. 2017). Recent studies have suggested that emergency granulopoiesis is essential for the protective effects of BCG-vaccination (Brook et al. 2020). However, the immunomodulatory effects of BCG-vaccination are multifactorial and include the expansion of multiple myeloid progenitor cells and an increased inflammatory and immunosuppressive response that often co-exist (Freyne et al. 2018; Kaufmann et al. 2018).

Although it is known that BCG-vaccination protects newborns from sepsis, little is known about the cellular or molecular events controlling BCG-induced trained immunity in myeloid cells, a primary target of innate immunity. Specifically, BCG’s effects on neonatal myeloid populations in general and myeloid-derived suppressor cells (MDSCs) in particular, remain unclear, especially since MDSC’s expand during pregnancy and are increased after birth (Gervassi et al. 2014). As in adults, neonatal MDSCs are immunosuppressive and may control inflammation during microbial colonization of gut and lungs in the first weeks of life. However, neonatal MDSC transcriptomics also show upregulation of antimicrobial genes suggesting a direct role in microbial control (He et al. 2018). Thus, it remains unclear whether BCG administration in the neonate involves myeloid enhancement of antimicrobial or anti-inflammatory organ injury-sparing activities, or both.

Here we have examined BCG vaccination on the murine neonatal response to polymicrobial sepsis. Although BCG vaccination was itself associated with a modest systemic inflammatory response and further expansion of splenic and blood MDSCs, the improvements in sepsis survival due to BCG vaccination were associated with widespread reductions in the systemic inflammatory response and altered composition of myeloid precursors and effector cell subsets, and their transcriptional response.

## Materials and methods

### Mice

All studies were approved by the Institutional Animal Care and Use Committee at the University of Florida. Wild-type (C57BL/6j; B6) mice were purchased from Jackson Laboratories (Bar Harbor, ME) and were kept at the University of Florida’s rodent facility, where they received standard care, ad libitum feeding and 12-h light–dark cycle schedule. Mice were housed together for 1 week to allow their intestinal microbiome to acclimatize and then were kept in harem-breeding schemes or were used as cecal slurry (CS) donors (Wynn et al. 2007). Once a pregnant female was identified, it was separated and monitored daily to accurately record the date of birth of the pups. Mixed-sex litters were used for all experiments.

### BCG administration and polymicrobial sepsis induction

Neonatal mice from the same litter were randomized to reduce variation from differences in maternal care and virulence of different lots of CS. Pups received either 10^5^ CFU of the BCG vaccine (TICE® strain, Merck, Rahway, NJ), 100 μg of the heat-inactivated BCG vaccine or saline, subcutaneously either at birth (P1) or on day 4 of life (P4). Polymicrobial intra-abdominal sepsis was induced in neonatal mice at day 7 of life (P7) using the CS model as described previously (Wynn et al. 2007). Briefly, cecal contents were harvested from a wild-type female adult mouse and an 80 mg/ml of fresh CS solution was prepared using 5% dextrose. CS (1.1–1.3 mg/g BW) was administered intraperitoneally to achieve the desire lethality (LD_30–70_). After sepsis-induction, mice were monitored every six hours for the first 48 h, then every 12 h thereafter, for any pre-determined IACUC-approved signs of being moribund (scattering of the pups, absence of milk spot and hypothermia) (Wynn et al. 2007; Starr et al. 2014). Survival was assessed for seven days following sepsis induction. Moribund animals were euthanized following approved IACUC guidelines and were considered non-survivors. Additional neonatal mice were used to collect blood and spleen immediately prior to the induction of sepsis and at six- and 18- hours following sepsis induction. For single cell transcriptomic analyses, we also used naive and septic, young adult (14–16 weeks) B6 mice as reference to aid in cluster assignment/identification (n = 8–10 per group).

Sepsis in adult mice was induced using the cecal ligation and puncture (CLP) model followed by daily chronic stress (CLP + DCS) for seven days, as previously described (Stortz et al. 2019). Briefly, a laparotomy was performed under isoflurane anesthesia, the cecum was isolated, and approximately 0.5 cm of cecum was ligated below the ileocecal valve and punctured through and through with a 25-gauge needle. Analgesia, fluid resuscitation and antibiotics were provided for 72 h. One day after CLP, mice were placed into a restraint holder for two hours daily for seven days to induce DCS. Seven-days post CLP + DCS (CLP + DCS7 d), animals were euthanized to collect their spleen.

BCG vaccine is indicated for the prevention of tuberculosis (TB) in infants and children who are at high risk for TB exposure. CDC guidelines state that BCG-vaccination is not generally recommended for use in the U.S. because of the low risk of infection with *M. tuberculosis* and the variable effectiveness of the vaccine against the adult pulmonary disease. To overcome the potential risks of BCG-vaccination in neonates, heat-inactivated BCG (HI-BCG) vaccine was used to study its role in experimental murine neonatal sepsis. BCG vaccine (TICE^®^ strain, Merck, Rahway, NJ) was heat-inactivated by the Division of Infectious Diseases and Global Medicine, University of Florida Emerging Pathogens Institute. Briefly, BCG vaccine was diluted following the manufacturer’s instructions and incubated in a water bath at 80 °C for 20 min. To assess bacteria growth, 100 μl of the heat-inactivated suspension was transferred to Lowenstein-Jensen medium and incubated at 37 °C for 8 weeks.

### Systemic cytokine quantification

Following euthanasia, neonatal mice blood was collected in heparinized tubes by decapitation. After centrifugation (1,500 × *g* for 10 min), plasma was collected and cytokine concentrations were quantified by a customized mouse Luminex® discovery assay (R&D systems, Minneapolis, MN), including: KC (CXCL1), TNF, IFNγ, S100 A9, IL- 6, IL- 10, FGF2 (FGF basic), RANTES (CCL5), G-CSF, GM-CSF and MIP- 2 (CXCL2).

## T-cell isolation and proliferation assays

Naive adult T cells were isolated from spleens using a mouse T cell isolation kit (Stemcell Technologies, Vancouver, CA), following the manufacturer’s instructions. Briefly, B6 adult mice (T-cell donors) were euthanized by CO_2_ asphyxiation followed by cervical dislocation, spleens were removed aseptically, and single cell suspensions were made using 70 μm filters. Isolated T-cells were labeled with cell trace violet (Thermo Fisher Scientific, Waltham, MA) at 37 °C for 15 min. Following incubation, 1 × 10^5^ T lymphocytes were seeded into a 96-well plate and stimulated with soluble anti-CD3/CD28 antibodies (BD Biosciences, Franklin Lakes, NJ) or culture media (DMEM, 2 mM L-glutamine, 50 μM mercaptoethanol, 10 mM HEPES, 50 μg/ml gentamycin) for unstimulated wells (control). Splenic CD11b^+^Gr1^+^ cells were isolated from naive, BCG-treated, septic and BCG-treated plus septic neonatal mice using EasySep^™^ mouse MDSC (CD11b^+^Gr1^+^) isolation kit (Stemcell Technologies, Vancouver, CA) and were co-cultured with stimulated T cells (2:1 ratio) at 37 °C and 5% CO_2_ for four days. Due to the small size of the spleens and relatively few cell numbers, spleens from three similar neonatal mice were pooled for a single measurement. Cells were then stained with anti-mouse CD8-PE and anti-mouse CD4-APC (BD Biosciences, Franklin Lakes, NJ) and analyzed via flow cytometry using a ZE5 Cell Analyzer (Bio-Rad Laboratories, Hercules, CA). Proliferation index was calculated as the total number of cell divisions divided by the number of cells that went into division (considering cells that underwent at least one division) (Barrios et al. 2024).

### Metabolic and extracellular flux analyses

Oxygen consumption rate (OCR) and extracellular acidification rate (ECAR) from splenic CD11b^+^Gr1^+^ cells were assessed using an Agilent Seahorse XF96^™^ Analyzer (Agilent Technologies, Santa Clara, CA). Briefly, 5 × 10^5^ splenic CD11b^+^Gr1^+^ cells pooled from three pups were added to a poly-L-lysine pre-coated plate in 180 μl Seahorse RPMI medium supplemented with 10 mM glucose and 2 mM glutamine (pH 7,4) and centrifuged at 1,000 × *g* for 5 min. Cell culture plate and utility plate with sensor cartridge were equilibrated after incubation in a non-CO_2_ 37 °C incubator for one hour. The different inhibitors of the Seahorse XF Cell Glycolytic Rate Assay™ kit (Agilent Technologies, Santa Clara, CA) were added to the corresponding ports of the sensor cartridge prior to starting the assay. Therefore, 20 μl of 5 μM rotenone and 5 μM antimycin A solution were added to port A and 22 μl of 500 μM 2-deoxyglucose (DG) solution to port B. After calibration, cell respiration parameters were determined by stepwise injection of the different inhibitors. During each measurement cycle, OCR and ECAR were determined three times involving three minutes of mixing and three minutes of measurement.

### Splenic myeloid cell phenotyping and quantification

Spleens were collected prior to sepsis (on day of life 6; P6) and six and 18 h after CS injection. To prepare samples for flow cytometry analysis, splenocytes were resuspended in ACK^™^ buffer (Thermo Fisher Scientific, Waltham, MA) to lyse red blood cells. After washing with PBS, the cells were resuspended in a cocktail of antibodies targeting innate immune effector cells and hematopoietic stem (HSCs) and progenitor (HPCs) cells. The following antibodies obtained from BD Biosciences (Franklin Lakes, NJ) and Biolegend (San Diego, CA) were used to prepare three main cocktail mixes (Supplementary key resources table): I-A/I-E (MHC-II)-AF700, F4/80-PE, CD11b-PECy7, Ly6G-APC, Ly6 C-FITC. For hematopoietic stem and progenitor cells (HSPCs) analysis, splenocytes were stained with anti-mouse: FITC-Lineage cocktail (Lin- 1), PerCP-CD117 (c-kit), PE-CD34, Bv421-CD135 and APC-CD127 (IL- 7Rα). All fluorochrome-labeled antibodies for HSPC analysis were obtained from Biolegend (San Diego, CA). After staining for 15 min at 4 °C in the dark, cells were washed with PBS and analyzed on a ZE5 Cell Analyzer (Bio-Rad Laboratories, Hercules, CA) followed by FlowJo^™^ v10.8 Software (BD Biosciences, Franklin Lakes, NJ) analysis. In order to quantify neonatal splenic MDSCs using flow cytometry, CD11b^+^MHCII^−/low^F4/80^−^ cells were initially gated, then further delineated by Ly6 C and Ly6G expression to yield MDSC subsets: M (monocytic)- (CD11b^+^ MHCII^−/low^F4/80^−^Ly6G^−^Ly6 C^+^) or PMN (polymorphonuclear or granulocytic)- (CD11b^+^MHCII^−/low^F4/80^−^Ly6G^+^) MDSCs. All other splenic myeloid cells were quantified as monocytes (CD11b^+^Ly6G^−^Ly6 C^+^), neutrophils (CD11b^+^Ly6G^+^) and macrophages (CD11b^+^F4/80^+^). Fluorescence minus one (FMO) samples were included to identify and gate the cells (Figure S3 A). Compensation was performed using compensation beads (BD Biosciences, Franklin Lakes, NJ).

### Single-cell RNA sequencing (scRNA-seq) analysis

#### Single cell collection

Splenocytes from naive, BCG-vaccinated, septic, and BCG plus sepsis (eighteen hours post-CS) neonatal mice, were obtained after mechanical dissociation using 70 μm strainers. After centrifugation at 300 × *g* for 10 min, red blood cells (RBC) were lysed with ACK lysing buffer (Thermo Fisher Scientific, Waltham, MA) for five minutes at room temperature. Total splenocytes were washed in PBS supplemented with 2% FBS, quantified (Cellometer Auto 2000 Cell Viability Counter; Nexcelom Bioscience, Lawrence, MA) and diluted to 10^6^ cells/ml before single-cell library preparation. Libraries were prepared with Chromium Next GEM Single Cell 5’ v2 kit targeting 10,000 cells/lane on the 10 × Chromium microfluidics device (10 × Genomics, Pleasanton, CA). Libraries were sequenced at a minimum depth of 20,000 read pairs per cell on an Illumina Novaseq^™^ X plus platform (Illumina, San Diego, CA).

#### Data pre-processing

Cell Ranger Single-Cell Software v 7.0.1 (10 × Genomics) was used to obtain FASTQ files from the Illumina base call files, align the sequencing reads to the mm10 mouse transcriptome reference using “intronic reads included” mode, and further quantify the expression level of transcripts in single-cells.

#### Quality control and clustering

Subsequent analyses were performed using R software v 4.3.2 and Seurat package v 5.0.1 (Hao et al. 2024). Low-quality cells (cells expressing > 10% mitochondrial transcripts or number of transcripts < 200) and doublets (number of transcripts > 6000) were filtered out. Gene expression data was natural-log normalized. The top 2000 high-variability genes were selected using the ‘FindVariableGenes’ function. The gene expression data were then scaled and subjected to dimensionality reduction through Principal Component Analysis (PCA). To integrate cells from all samples, PCA embeddings were corrected using the Harmony package, version 1.2.0 (Korsunsky et al. 2019). For unsupervised clustering, the 30 nearest neighbors were identified by function ‘FindNeighbors’ based on Harmony adjusted PCA embedding. ‘FindClusters’ functions were further applied to identify cell clusters based on the Louvain algorithm (Blondel et al. 2008). The identified clusters were visualized in Uniform Manifold Approximation and Projection (UMAP) embeddings which were calculated using the top 30 principal components.

#### Cell type annotation

To accurately annotate cell types in the neonatal murine model, data from the more extensively studied young adult murine model was employed as a reference. This involved integrating neonatal sample data with young adult samples, which included samples from both healthy and septic animals. Cell types were manually annotated based on marker genes identified from the literature. Initially, broad cell categories such as myeloid, lymphoid, DC and plasma cells were determined. Once these major groups were established, the myeloid cell compartment was further divided into subsets, following the unsupervised clustering process shown above within each cell cluster.

#### Differential expression analysis

For each cell type of interest, differential expression (DE) analysis across groups were performed using non-parametric Wilcoxon rank sum test with p-values adjusted by Bonferroni correction. Genes were identified as differentially expressed (DEGs) based on two criteria: an adjusted p-value of less than 0.001 and an absolute log_2_ fold change (FC) greater than 1. Marker genes were identified for each cell type by comparison to all others.

#### Trajectory analysis

RNA trajectory analysis was performed using Python 3.10.13 and scVelo v 0.3.1 (Bergen et al. 2020), based on the pre-mature (unspliced) and mature (spliced) transcript information, which were obtained by Velocyto v 0.17.17 (Manno et al. 2018). *S*ubsequently, gene selection focused on the top 2,000 genes exhibiting high variability and expression in at least 20 cells, followed by their log_2_ normalization. The analysis employed the top 30 principal components to identify the 30 nearest neighbors. Gene-wise RNA velocity was inferred using a stochastic velocity model. These estimations were further visualized in a UMAP embedding space derived from prior Seurat analysis. To identify pivotal genes impacting velocity, a differential velocity t-test was applied on the estimated velocity features (i.e. velocity score, velocity length and directionality across identified cell subtypes) of each gene.

### Pathway enrichment analysis

Enriched canonical pathways were identified using Ingenuity Pathway Analysis (IPA QIAGEN Inc. Germantown, MD, https://digitalinsights.qiagen.com/IPA) using Fisher’s exact test and adjusted with the Benjamin-Hochberg correction. Only genes whose expressions significantly changed (FDR, q < 0.001 and onefold log_2_ change) were used for the analysis. Significance was determined using the activation z-score (z-score ≥ 2.00 refers to predicted activation and z-score ≤ − 2.00 refers to predicted inhibition for particular functions).

### Statistical analysis

All other statistical analyses were performed using GraphPad Prism software v 10 (Boston, MA). Data were summarized as mean ± standard error of the mean (SEM) and medians and IQR followed by one-way ANOVA or Kruskal–Wallis test for those variables that did not meet normality assumptions (Shapiro–Wilk test) of one-way ANOVA. Kaplan–Meier survival analysis was used to examine the impact of BCG vaccination on outcome. Two-tailed p values less than 0.05 were considered as having statistical significance.

## Results

### BCG-vaccination at birth improves survival to murine neonatal polymicrobial sepsis

**Fig. 1:**
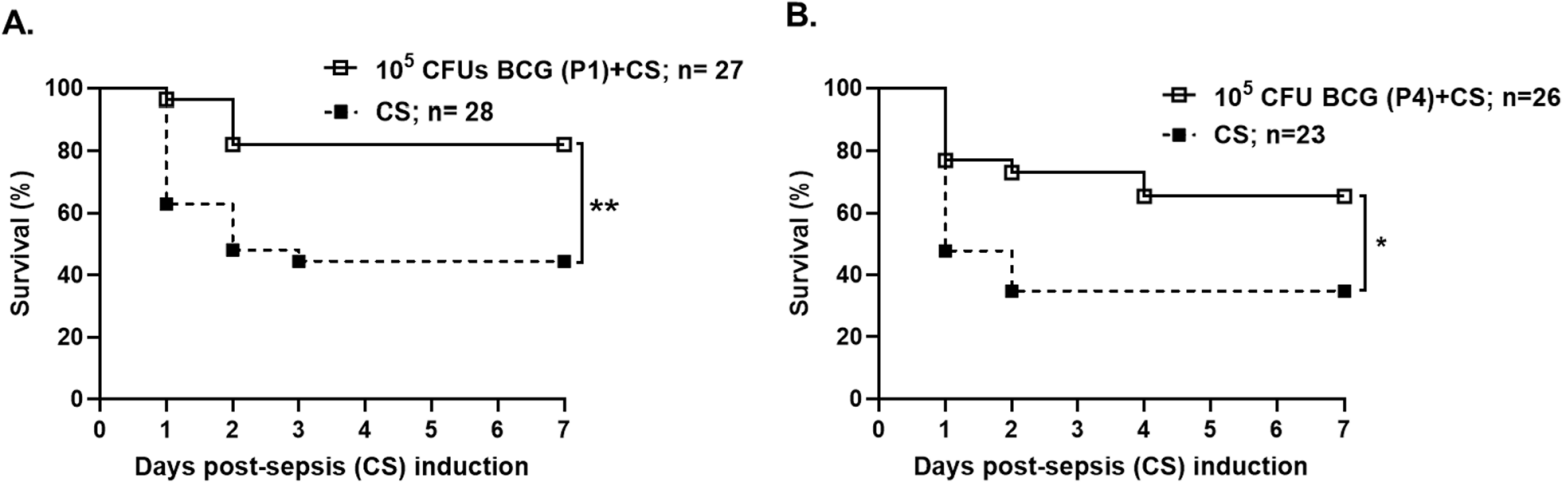
BCG-vaccination increases survival following intrabdominal poly-microbial sepsis in neonatal mice. We analyzed the effect of BCG-vaccination either **A** at birth (P1) or **B** at day of life 4 (P4) in sepsis mortality induced by cecal slurry (CS) on day of life 7 (P7). Kaplan–Meier survival curves estimated over the lifespan after sepsis induction. Survival data were compared via the Log-rank test. **p < 0.01; *p < 0.05

It has been previously demonstrated that both aluminum-based and attenuated bacterial immunoadjuvants improve survival in murine neonatal sepsis (Brook et al. 2020; Rincon et al. 2018). To investigate the role of immunoadjuvant administration early in life and trained immunity on the mechanisms of improved sepsis outcomes, neonatal mice received either live-attenuated BCG vaccine or HI-BCG at birth (P1) or at day 4 of life (P4) prior to sepsis induction at day 7 of life (P7). The greatest survival benefit following sepsis was observed with BCG administration at birth (82% versus 44%, p < 0.01; Fig. 1A). Survival benefit was also significantly improved with BCG administration at P4, albeit at a slightly lower rate compared to day 1 (65% versus 35% sepsis; Fig. 1B). Heat-inactivated BCG administration did not provide any survival benefit regardless of the administration time (Figure S1). No complications related to BCG-vaccination were observed, other than a blister at the site of injection 5–7 days post-vaccination.

### BCG induces pro-inflammatory cytokine production prior to sepsis induction

**Fig. 2:**
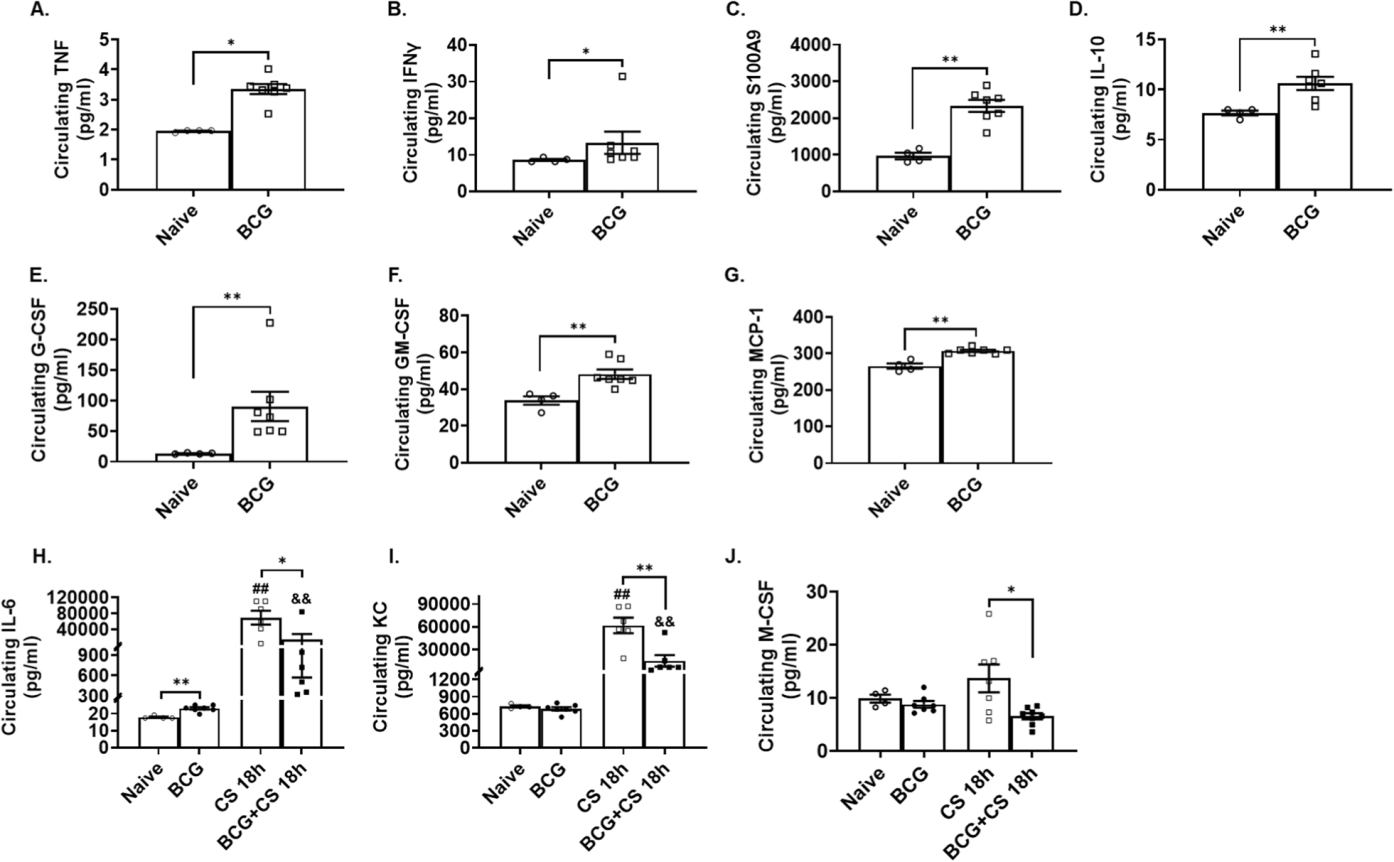
Effect of BCG-vaccination on plasma cytokine/chemokine levels after neonatal sepsis induced by cecal slurry (CS). Neonatal mice received either BCG or saline at birth and 7 days later were challenged with intra-abdominal polymicrobial sepsis. Blood was collected prior to- (Naive or BCG; panels **A**–**G**) and eighteen hours (CS 18 h) post-sepsis induction (panels **H**–**J**). Changes in concentration of circulating cytokines and chemokines were quantified in Naive or BCG-vaccinated (BCG), sepsis (CS 16 h vs. BCG + CS 18 h). n = 4–7 per group per time point. Bars represent median and interquartile range. **p < 0.01; *p < 0.05. ##p < 0.01 vs. Naive; &&p < 0.01 vs. BCG

Based on the survival benefit to sepsis seen in the day one BCG-vaccinated neonatal mice (P1), the potential effects of BCG on the systemic inflammatory response immediately prior to and after sepsis induction were evaluated by measuring multiple plasma cytokine concentrations. First, we assessed the systemic pro-inflammatory response to neonatal sepsis, via quantification of circulating levels of key cytokines (TNF, IFNg, IL- 6, S100 A9, IL- 10, bFGF), chemokines (KC, RANTES, MCP- 1) and growth factors (G-CSF, GM-CSF) (Figure S2). While BCG-vaccination induced a modest systemic cytokine and chemokine production in itself (p < 0.05 versus naive neonatal mice; Fig. 2A–H), the plasma cytokine response to sepsis was more attenuated in the BCG-treated mice. BCG-pretreatment significantly reduced circulating IL- 6, KC, and M-CSF levels 18 h post-sepsis induction compared to unvaccinated septic neonatal mice (p < 0.05 vs. CS 18 h; Fig. 2H–J).

### BCG induces splenic hematopoietic stem cell and myeloid cell expansion

To further explore whether BCG-vaccination modulates other aspects of host protective immunity in neonatal mice, we examined whether BCG’s beneficial effect on neonatal sepsis outcome might be explained by reprogramming immune effector cell expansion (Netea et al. 2016; Andualem et al. 2023). Not surprisingly, we found that splenic neutrophil (Ly6G^+^CD11b^+^), monocyte (Ly6 C^+^CD11b^+^) and macrophage (F4/80^+^CD11b^+^) numbers were significantly increased in mice treated with BCG prior to sepsis (p < 0.05 versus naive; Figure S3 B-C) and post-sepsis induction (p < 0.05 versus unvaccinated septic pups; Figure S3 B-C), as previously reported (Brook et al. 2020). Interestingly, BCG-vaccination reduced the percent of inflammatory splenic monocytes (Ly6 C^hi^CD11b^+^) compared to naive neonatal mice prior to sepsis (p < 0.01; Figure S3 C). In contrast, by 18 h after sepsis, splenic neutrophil, monocyte and macrophage numbers were similarly depleted in both naive and BCG-vaccinated pups, consistent with a rapid exodus from the spleen (Figure S3 C).

**Fig. 3:**
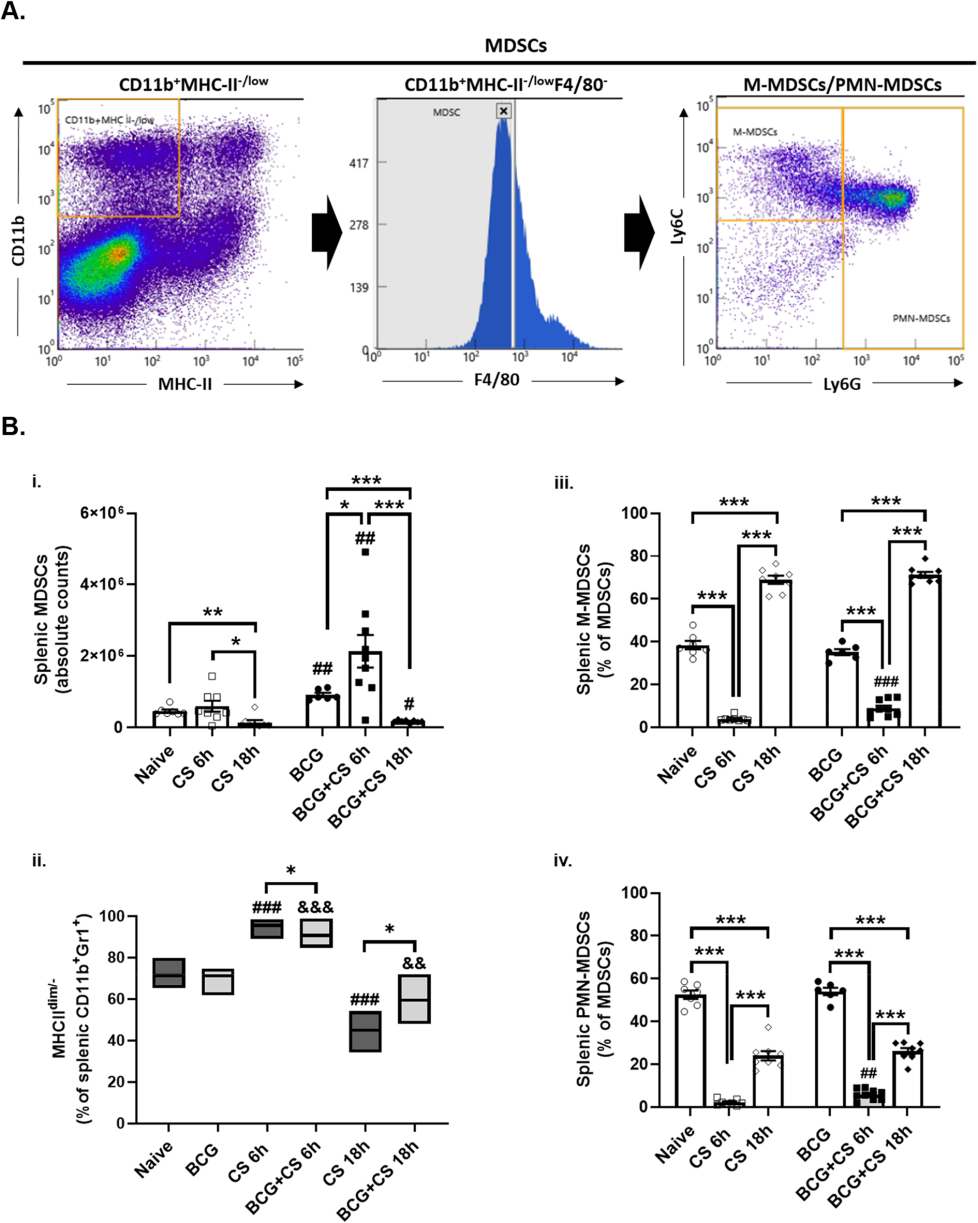
Effect of sepsis and BCG-vaccination on splenic CD11b^+^Gr1^+^ cell numbers. Neonatal mice received either BCG or saline at birth and 7 days later were challenged with intra-abdominal polymicrobial sepsis (CS). **A** Phenotypic characterization of splenic CD11b^+^Gr1^+^. Murine neonatal splenocytes were collected prior to- (Naïve or BCG-vaccinated [BCG]), six (CS 6 h vs. BCG + CS 6 h) and 18 h CS 18 h vs. BCG + CS 18 h) after sepsis. **B** Splenocytes were stained with fluorochrome-conjugated antibodies to quantify the expression of (i) CD11b^+^Gr1^+^ cells using flow cytometry. (ii) MHCII expression on CD11b^+^Gr1^+^ cells. (iii) Percent of CD11b^+^Gr1^+^ cells expressing Ly6 C and (iv) Ly6G markers. n = 6–7 per group per time point. Error bars indicate SEM. ***p < 0.001; **p < 0.01; *p < 0.05. ##p < 0.01 and #p < 0.05 vs. unvaccinated neonatal mice

CD11b^+^Gr1^+^ cells naturally accumulate in the spleen and secondary lymphoid organs during the neonatal period (He et al. 2018). Immature myeloid cells represent subsets of this more general CD11b^+^Gr1^+^ population that have lowered surface expression of MHC class II (MHCII^dim/−^), and have been termed, MDSCs (Ko et al. 2009). BCG-vaccination induced a significant expansion of these cells prior to- and six- hours following sepsis induction (p < 0.01; Fig. 3). The expansion of MDSCs was transient; however, by eighteen hours post-CS MDSC numbers had declined to below baseline numbers. However, these reduced MDSC numbers at 18 h were still significantly higher in septic pups that received the BCG vaccine at birth, compared to their septic unvaccinated littermates (p < 0.05; Fig. 3B). Interestingly, while at six hours post-CS, most MDSCs were Ly6 C-Ly6G- double negative cells, at 18 h after sepsis most MDSCs were M-MDSCs (Fig. 3B).

To examine whether BCG-induced expansion of more differentiated splenic myeloid subsets was due to enhanced myelopoiesis, we quantified hematopoietic stem cells (Lin^lo/−^Sca- 1^+^c-kit^hi^ [LSKs]) and myeloid cell progenitors (common myeloid progenitors [CMPs] and granulocytic-monocyte progenitors [GMPs]) in the spleen, a hematopoietic organ in the neonate (Bronte and Pittet 2013). BCG-vaccination caused a significant increase in both splenic LSK and GMPs (p < 0.01 and p < 0.05 versus naive, respectively; Figure S4 A-B), as well as an increase, albeit not significant, in CMPs (Figure S4 B). Following sepsis, we observed significant depletion of splenic CMPs and GMPs in both naive and BCG-vaccinated mice at 18 h (p < 0.05 versus naive); LSK numbers were not different after sepsis between naive and BCG-vaccinated mice at 18 h post sepsis, although compared to pre-sepsis, had declined significantly in vaccinated mice (Figure S4).

### BCG-vaccination alters the CD11b^+^Gr1^+^ functional responses to sepsis

#### T cell proliferation

**Fig. 4:**
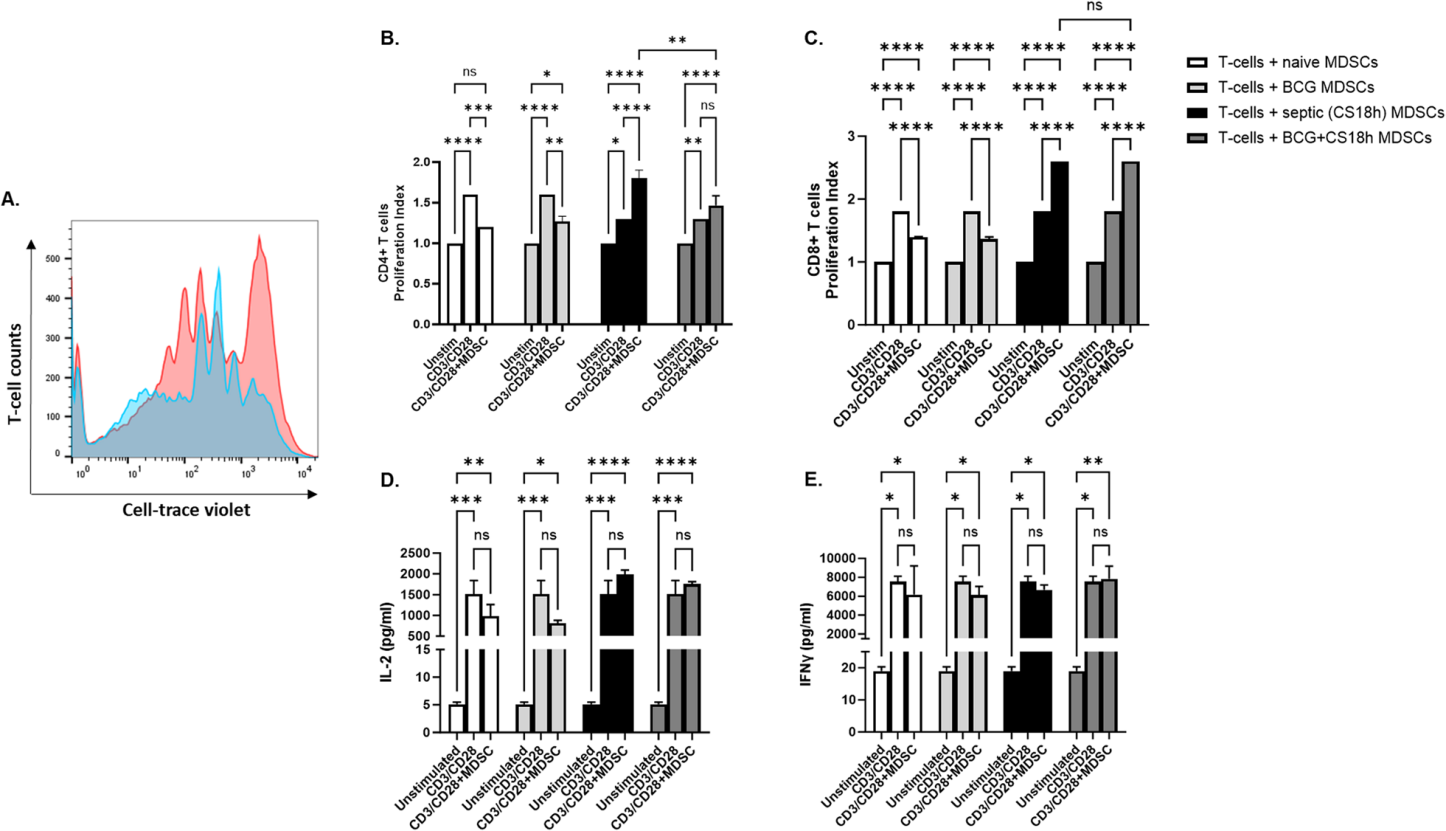
In vitro T-cell proliferation index and cytokine production. **A** Representative histogram illustrating CellTrace violet dilution as a measure of adult T-cell proliferation cultured alone or with syngeneic neonatal splenic CD11b^+^Gr1^+^ cells, upon activation with anti-CD3/CD28 antibodies for 96 h. **B** Proliferation of CD4^+^ and **C** CD8^+^ T cells unstimulated, stimulated (T-cells plus anti-CD3/CD28) and co-cultured with splenic neonatal either naive, septic or BCG plus septic CD11b^+^Gr1^+^ cells. **D**, **E** IL- 2 and IFNγ production in supernatants collected from T-cell proliferation assays. Kruskal–Wallis test was used for analysis of medians between groups. Error bars indicate SEM. ****p < 0.0001; ***p < 0.001; **p < 0.01; *p < 0.05

As BCG-vaccination reduced pro-inflammatory cytokine concentrations and induced expansion of splenic CD11b^+^Gr1^+^, additional functional changes in these cells were also examined. Subsets of CD11b^+^Gr1^+^ cells with low expression of MHCII have been considered to be MDSCs, as defined by their reduced antigen presentation and T-cell activation (Bronte and Pittet 2013). As expected, neonatal CD11b^+^Gr1^+^ splenocytes isolated from both naive and BCG-vaccinated mice, when co-cultured with T-cells from healthy young adult mice significantly suppressed CD4^+^ and CD8^+^ T-cell proliferation, compared to stimulated T cells alone (p < 0.01 vs. anti-CD3/28 wells; Fig. 4A–C). These findings suggest that the net balance of CD11b^+^Gr1^+^ cells from the neonatal spleen favor CD11b^+^Gr1^+^ cells immunosuppressive activity. Unexpectedly, coincubation of T cells with CD11b^+^Gr1^+^ splenocytes obtained 18 h after sepsis did not suppress ex vivo stimulated T-cell proliferation, regardless of whether these cells were isolated from BCG-vaccinated or naive mice (Fig. 4B, C). Rather, they stimulated T-cell proliferation. IL- 2 and IFNγ production in supernatants of T cell proliferation assays showed increased concentrations of both cytokines in the positive control wells (CD3/CD28); however, there was no difference in cytokine production when T cells were co-cultured with CD11b^+^Gr1^+^ cells from either naive, BCG-vaccinated or septic neonatal mice (Fig. 4D, E). These findings contradict what has been reported previously using a monomicrobial model of sepsis (Vance et al. 2021). Even though murine MDSCs have been historically defined as cells expressing both Gr1 and CD11b markers and given that myeloid cells (including MDSCs) are highly plastic, we cannot rule out the presence of other innate immune effector cells expressing these markers (e.g. PMNs, monocytes).

#### Oxidative metabolism

**Fig. 5:**
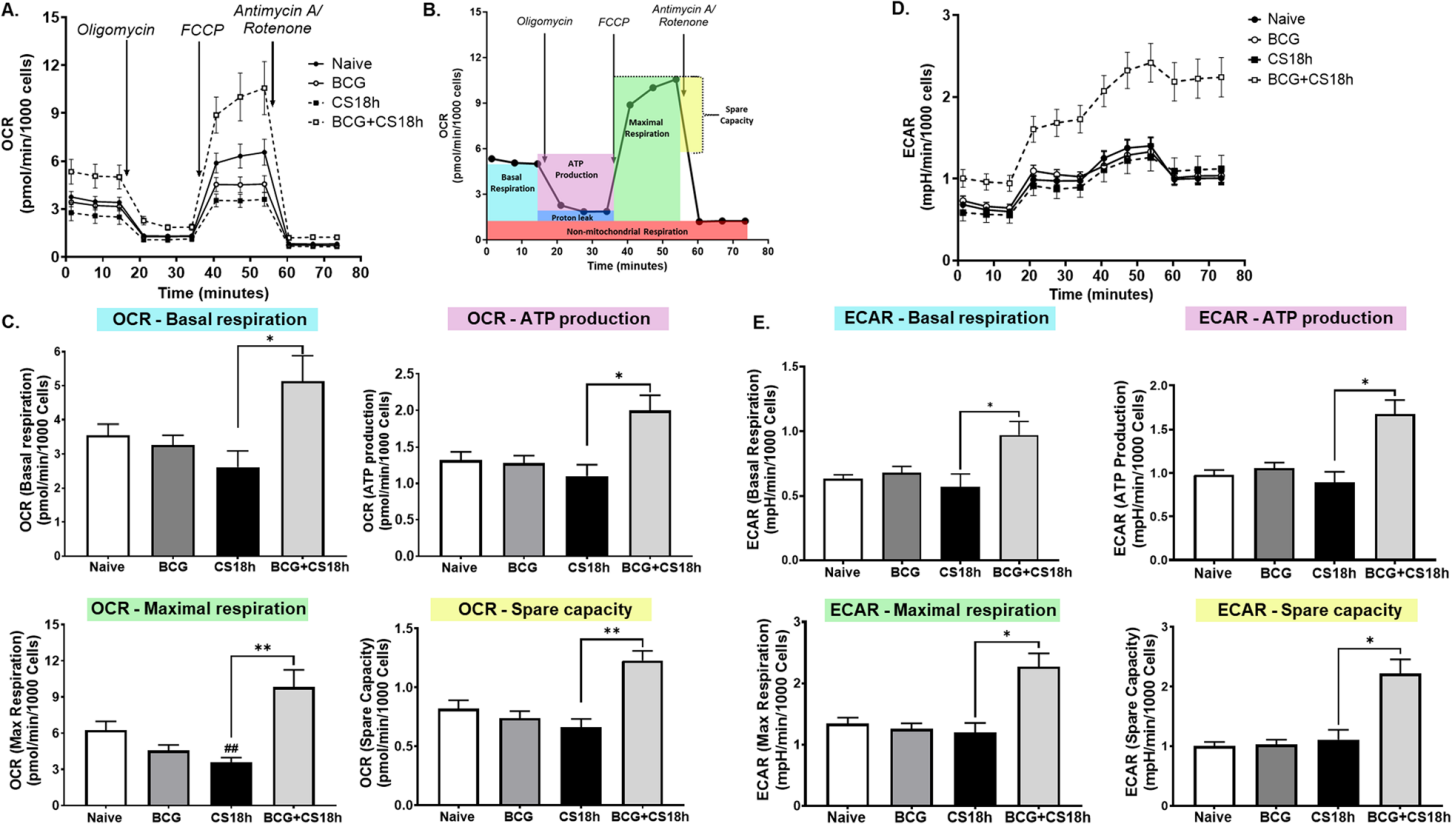
Mitochondrial respiration in naive, BCG-vaccinated, septic and BCG plus sepsis neonatal splenic CD11b^+^Gr1^+^ cells. **A** Splenic CD11b^+^Gr1^+^ isolated from BCG septic neonatal mice show increased oxygen consumption rate (OCR) and **D** extracellular acidification rate (ECAR). Mitostress test was performed by sequential addition of oligomycin (complex V inhibitor), FCCP (mitochondrial membrane depolarization), antimycin (complex III inhibitor) and rotenone (complex I inhibitor). Three neonatal mice were pooled per sample (n = 3/group). **B** A diagrammatic representation of mitostress test and functional significance of area under the curve. **C**, **E** Basal respiration, ATP production, maximal respiration and spare capacity. Error bars indicate SEM. **p < 0.01; *p < 0.05; ##p < 0.01 vs. Naive

Increasing evidence suggests that innate immune effector cells are able to modulate both their oxidative and glycolytic metabolism during periods of infection or stress (Puleston et al. 2017). Trained immunity, through epigenetic changes in host cells, is presumed to induce changes in oxidative metabolism that not only provide for increased energy demands, but also generate reactive oxygen and nitrogen species involved in both antimicrobial activities as well as suppression of T cell proliferation (Bhargavi and Subbian 2024). As inflammation is generally associated with a greater demand for ATP and higher metabolic activity, we therefore examined the dynamic changes in glucose utilization and mitochondrial function using a mitochondrial stress test on splenic CD11b^+^Gr1^+^ cells isolated from naive, BCG-vaccinated, septic or BCG-vaccinated plus septic neonatal mice. Quantitative analysis of mitochondria respiration (oxygen consumption rate, OCR) and extracellular acidification rate (ECAR) revealed that BCG-vaccination had no demonstrable effect on oxygen consumption or lactic acid production in isolated CD11b^+^Gr1^+^ splenocytes. The response after sepsis in the BCG-vaccinated pups, however, was dramatically different. Unlike that seen in unvaccinated animals (CS 18 h), BCG vaccination significantly enhanced mitochondrial oxidation and glycolysis on CD11b^+^Gr1^+^ splenocytes after sepsis (Fig. 5A–D).

### BCG induces transcriptional changes in myeloid cells

Functional and morphological adaptation of the innate immune response after BCG vaccination at birth represents de facto innate immune memory that leads to both an improved outcome to a secondary insult (sepsis), but also alterations in emergency myelopoiesis, CD11b^+^Gr1^+^ oxidative metabolism and systemic cytokine production. Each of these measurements, however, lack the cell specificity that can be obtained from single cell RNA sequencing. They also fail to demonstrate the breadth of the changes in the myeloid cell response to induction of trained immunity. We therefore conducted scRNA-seq analyses to determine myeloid cell changes associated with BCG-vaccination and trained immunity. scRNA-seq was performed in total splenocytes from either naive, BCG-vaccinated, eighteen-hours septic (CS 18 h) or BCG-vaccinated plus sepsis (BCG + CS 18 h) neonatal mice (n = 5–7 per group).

Since neonatal murine myeloid cell characterization at single cell level has not been fully resolved even in naive animals, we included transcriptomic analyses of splenocytes from young adult naive and septic mice as an additional reference cohort, especially to aid in the identification of MDSCs that are known to be significantly expanded in adult murine sepsis (Delano et al. 2007).

#### Single-cell transcriptome profile in neonatal splenic myeloid cells

**Fig. 6:**
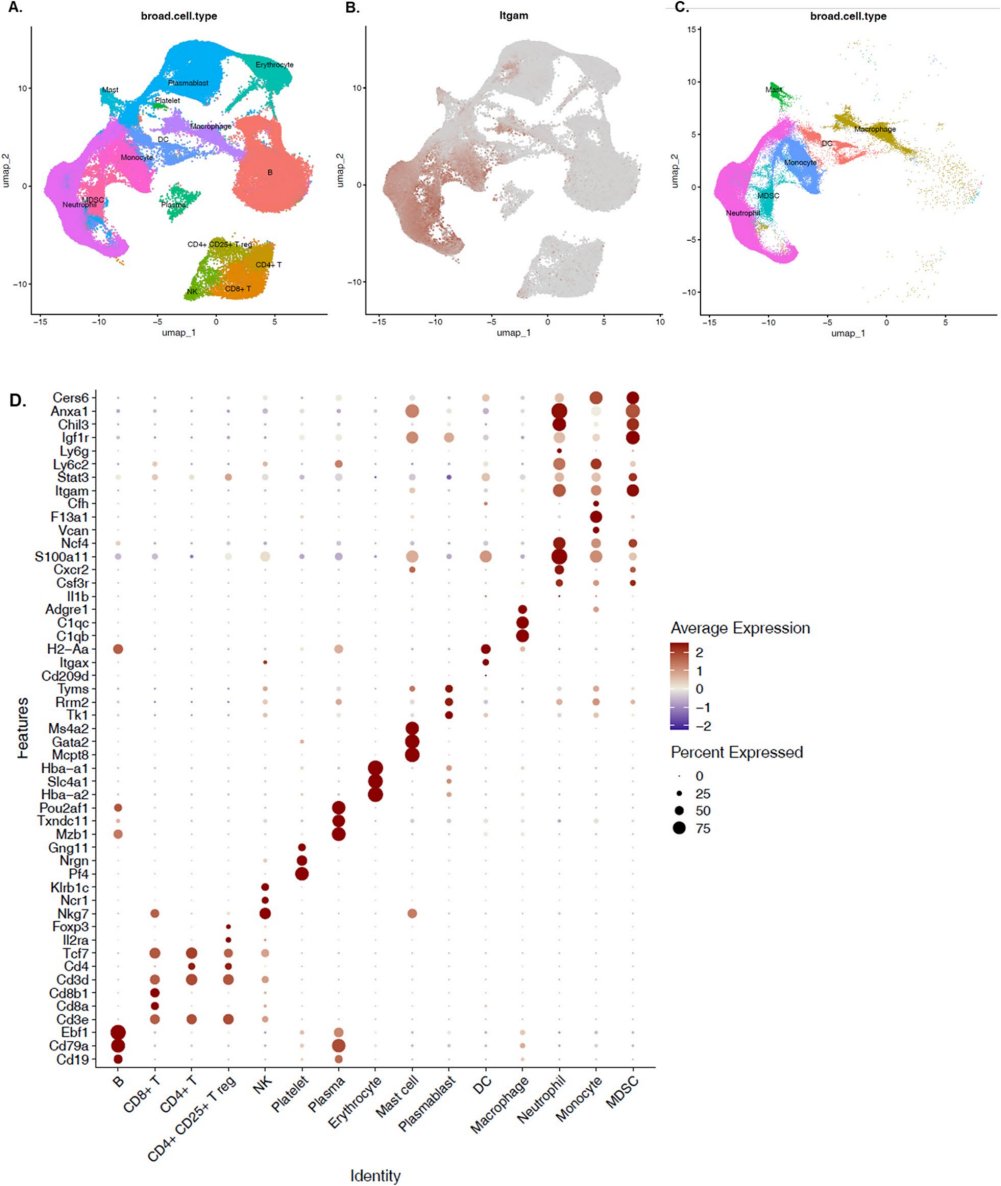

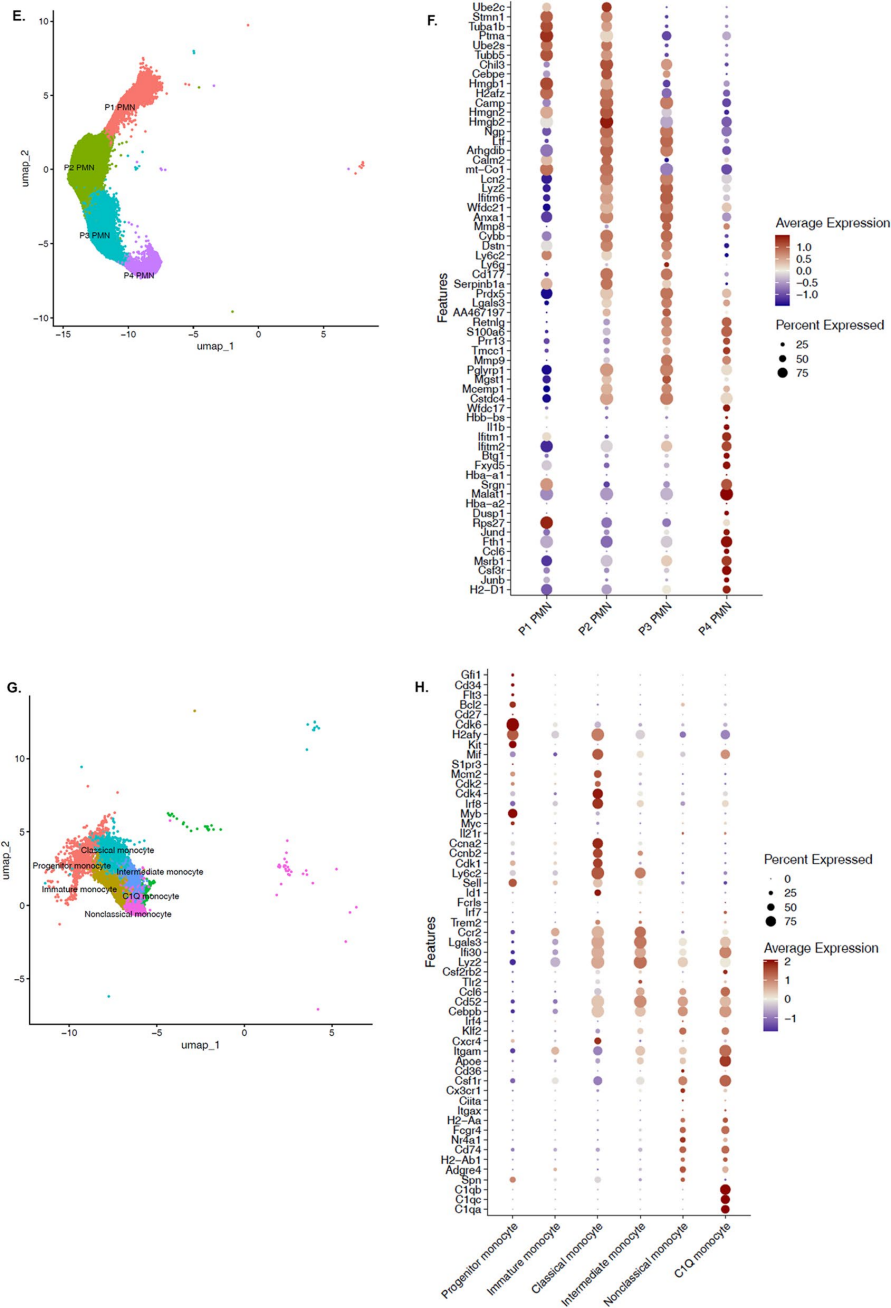
Categorization of myeloid cells in murine neonatal sepsis. **A** High quality cells from all groups were integrated into normalized dataset and subjected to PCS for dimensionality reduction and UMAP for visualization. Unbiased, graph-based clustering identified main myeloid cells clusters from splenocytes based on *Itgam* expression **B** and the expression of canonical gene markers (**C**, **D**); which further allowed annotation of PMNs (**E**, **F**) and monocytes (**G**, **H**) sub-clusters

In total we profiled 205,768 splenocytes (from all treatment groups, including neonatal and adults splenocytes) that provided a comprehensive analysis of myeloid cell heterogeneity. High quality cells from all groups were integrated into a normalized dataset and subjected to PCA for dimensionality reduction and UMAP for visualization (Fig. 6A). Cells with a myeloid cell signature (based on *Itgam* expression, Fig. 6B, C) and canonical lineage markers were selected for identification of main cluster cells and further analysis (Fig. 6D) (Grieshaber-Bouyer et al. 2021; Tabula Muris Consortium et al. 2018; Watanabe et al. 2024).

PMN gene markers (Grieshaber-Bouyer et al. 2021; Veglia et al. 2021) identified three immature granulocyte stages (P1 PMN to P3 PMN), which exhibited a recognizable correlation with neutrophil development (*Ube2c, Stmn1, Chil3, Camp, Ngp, Cd177, Anxa1, Cybb, Mmp8, Cebpe*) and identified a fully differentiated neutrophil cluster (P4 PMN), enriched for transcripts of chemokine receptors and genes associated with inflammation (*Il1b, Ccl6, Csf3r,*) (Fig. 6E, F) (Grieshaber-Bouyer et al. 2021). Similarly, clustering analysis of splenic monocytes yielded six subsets: non-classical (*Cx3cr1, Adgre4, Spn, B2 m, Cd74*), classical (*Ly6c2, Cxcr4, Ccr2,*), intermediate (*Ly6c2, Ccr2, Itgam*), C1Q^+^ (C1qb, C1qc, C1qa) immature and monocyte progenitors (*Gfi1, Cd34, Flt3*) (Fig. 6G, H).

Once most myeloid cells were annotated based on canonical gene markers, neonatal MDSCs were subsequently identified based on expression patterns from monocytic (M-) and granulocytic (PMN-) MDSC subsets that were identified in adult septic compared to adult naive mice. As MDSCs accumulate in several organs including bone marrow and spleen seven- and 14- days post-sepsis induction in adult mice (Delano et al. 2007), we chose this model (Figure S5 A) in order to identify in our experimental settings (i.e. neonatal mice) splenic MDSCs (Liu et al. 2021). First, by focusing our analysis on monocyte sub-clustering, we identified a unique monocyte subset (labeled as M-MDSCs) in adult septic mice (compared to naive) that was significantly enriched after sepsis (p < 0.001 versus naive adult mice; Figure S5 B-C). This cell type demonstrated 51 DEGs (CLP-induced sepsis versus naive, FDR q < 0.01 and abs.log_2_FC > 1), which had a predicted impact in downregulation of IL- 8 and focal adhesion kinase (FAK) signaling pathways. Similarly, we identified a cluster of PMN-like cells that exhibited a significant downregulation of pro-inflammatory genes including: *Fcgrt, Tnfrsf25, Ptgdr, Il12a, Il1b*, and these cells were significantly expanded seven days after CLP-induced sepsis in adult mice (p < 0.001 versus naive adult mice; Figure S5 B and S5 D). As expected, PMN- and M-MDSCs were rarely detected in naive adult mice, but abundant in septic animals (Figure S5 B-D), consistent with flow cytometric data reported previously (Delano et al. 2007).

#### Cell proportions of splenic MDSCs, monocytes and PMNs in polymicrobial neonatal sepsis

Once MDSCs were annotated, the effect of sepsis, BCG-vaccination or BCG-vaccination followed by sepsis induction on cell proportions were examined from neonatal splenocytes (18 h post-CS). As the greatest survival benefit to sepsis was observed when BCG was administered at P1, we continued our transcriptomic analyses in this experimental setting (BCG-vaccination at P1 followed by sepsis induction at P7). As expected (based on our flow cytometry data), naive neonatal mice had increased splenic M- and PMN- MDSC proportions compared to naive young adult mice (p < 0.01; Figure S6 A-C). BCG-vaccination only modestly induced the further expansion of M-MDSCs in healthy neonatal mice. Consistent with our findings using flow cytometry, BCG-vaccination induced the expansion of all monocyte subsets and terminally differentiated neutrophils (P4 PMN) prior to sepsis (Figure S7 A-D). Similarly, prior BCG-vaccination had no effect on MDSC proportions eighteen hours after induction of sepsis, compared to their unvaccinated littermates (Figure S6 B-C), which is compatible with our flow cytometry findings (Fig. 3 C-D). Conversely, BCG-vaccination increased the proportions of monocytes and PMNs in neonatal septic mice.

#### BCG-vaccination alters MDSC-associated transcriptomic patterns in septic neonatal mice

**Fig. 7:**
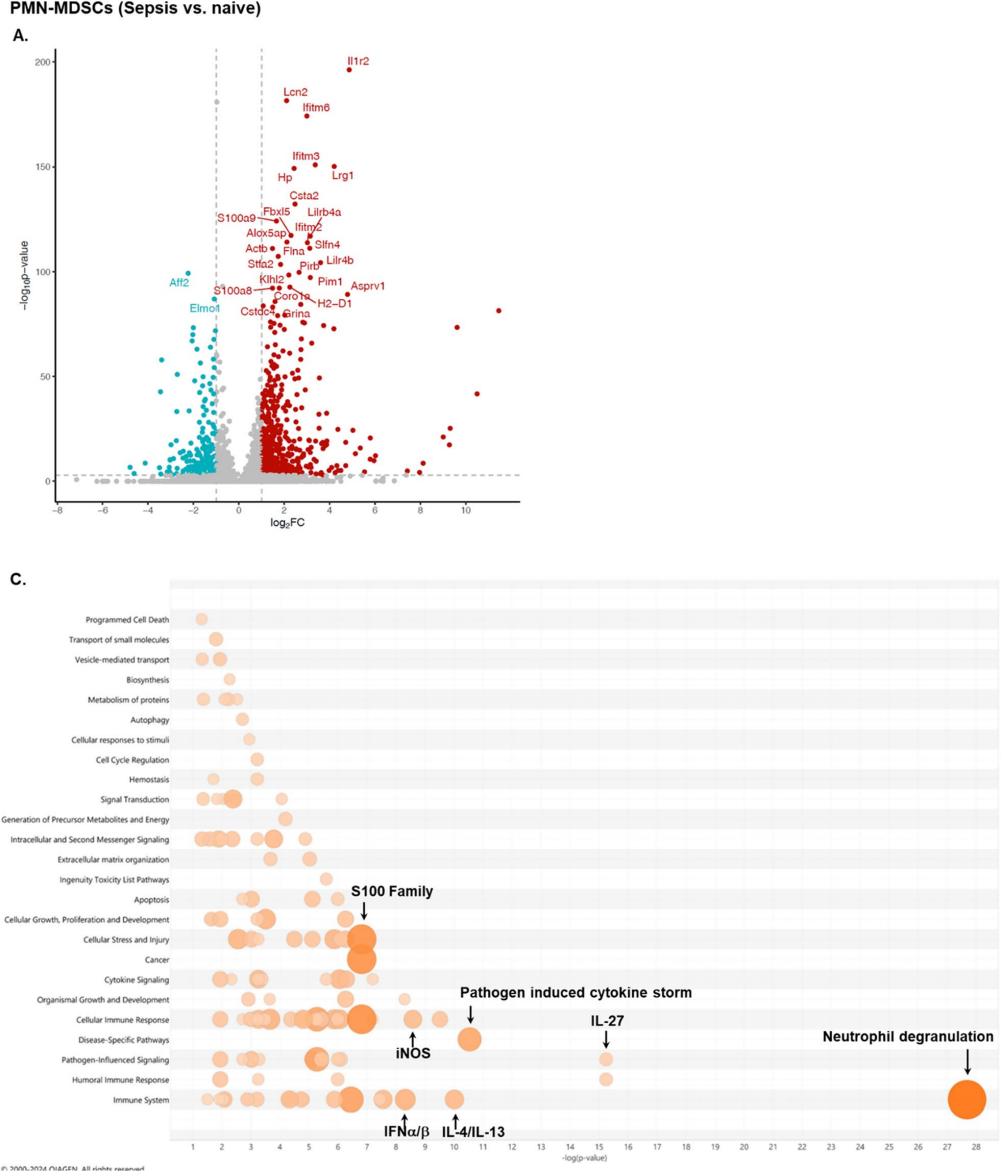

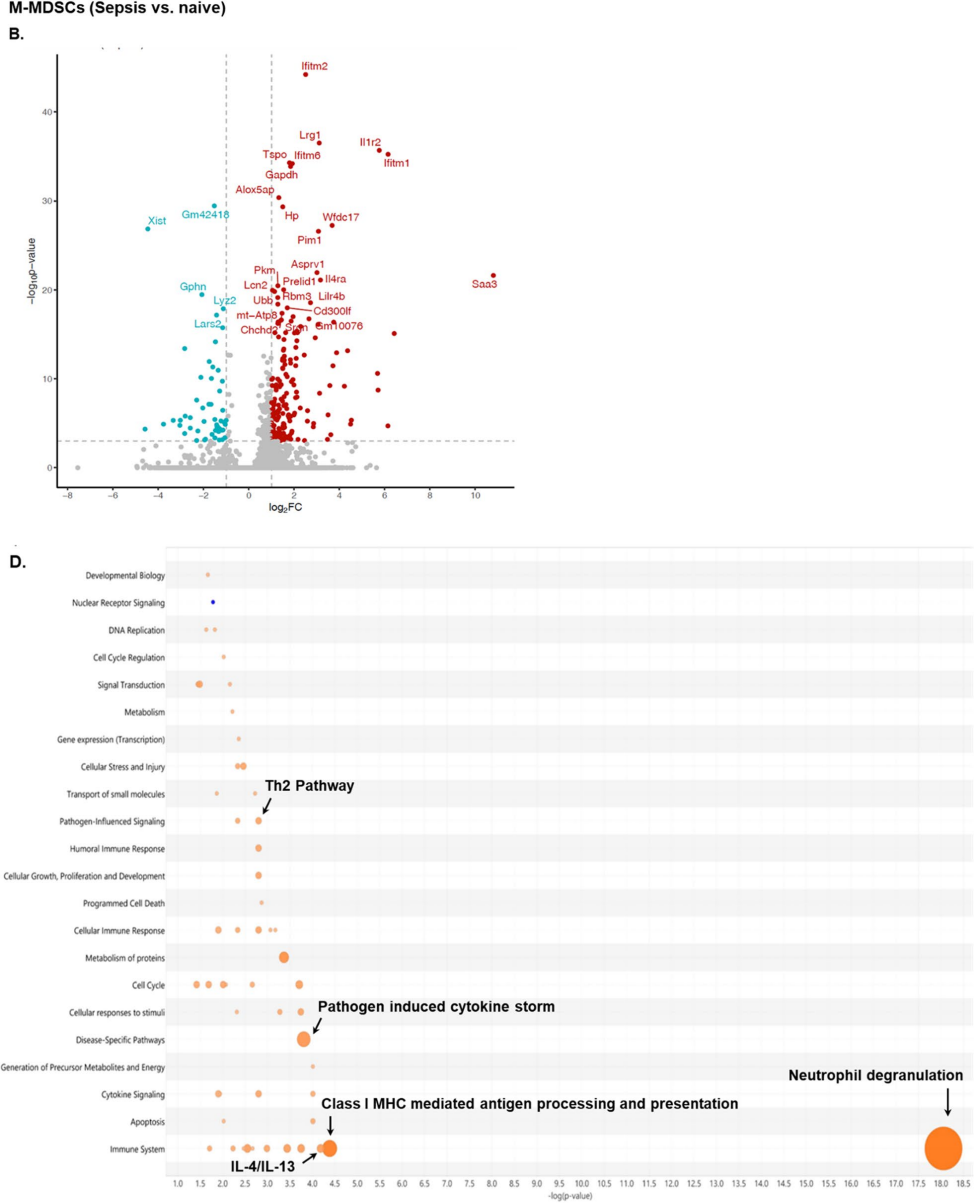

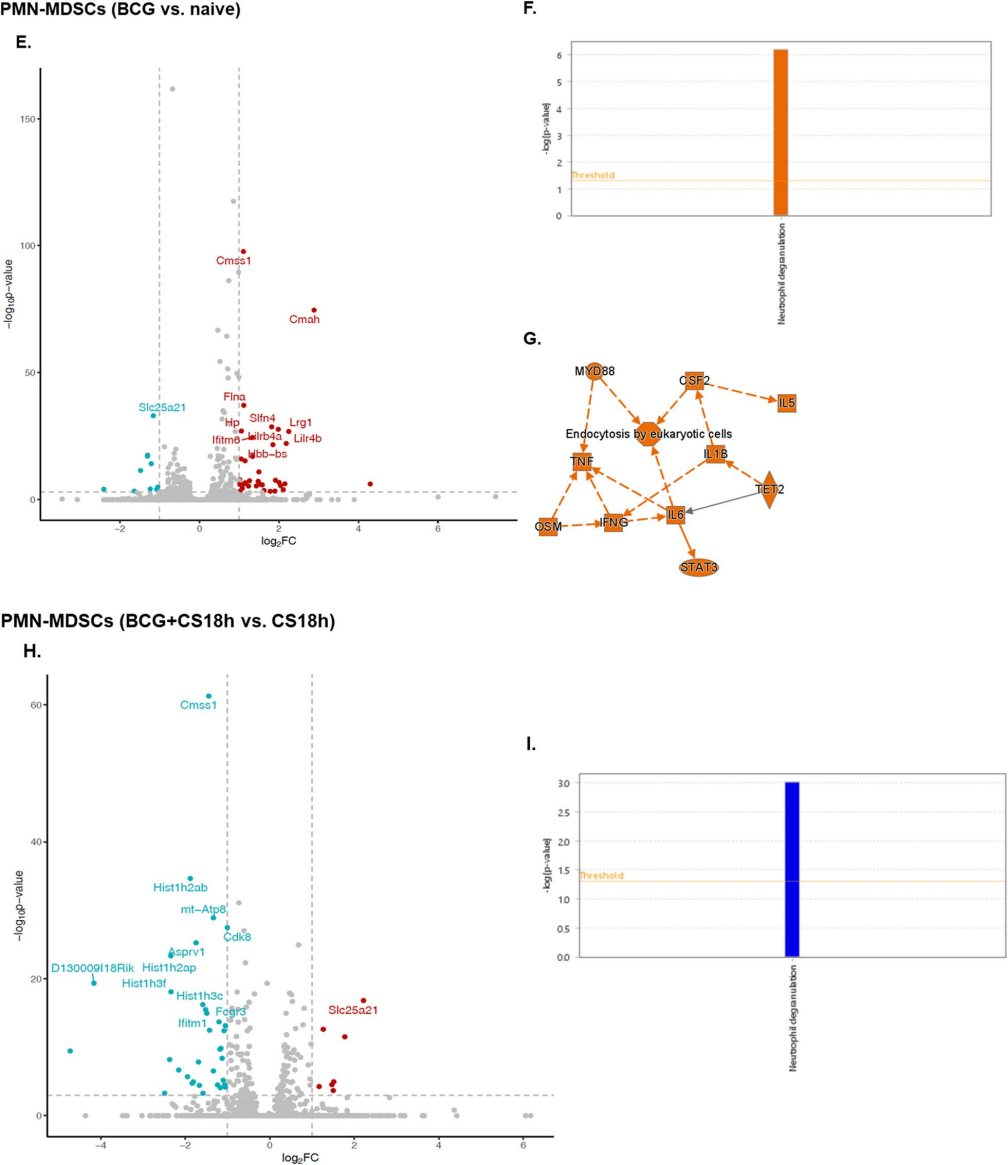
Differential expression of genes in splenic MDSCs in septic compared to naive neonatal mice (**A**–**D**) and effect of BCG-vaccination in neonatal MDSCs prior to (**E**–**G**) and after sepsis induction (**H**-**I**). **A**, **B** Volcano plots integrating *p-*values and log_2_FC for genes 18-h after sepsis-induced by cecal slurry (CS) in neonatal mice (compared to naive) in PMN-MDSCs and **B** M-MDSCs. **E**, **H** Volcano plots display DEGs in PMN-MDSCs of BCG-vaccinated versus naive neonatal mice and BCG followed by sepsis (BCG + CS18 h) compared to septic (CS18 h) neonatal mice. Top differentially expressed genes (DEGs) are labeled with the gene name. Red dots represent over-expression and blue dots down-regulation. Y-axis denotes –log10 p values while x-axis shows log_2_FC values. **C** Bubble chart of the top canonical pathways list generated by ingenuity pathway analysis (IPA) of PMN-MDSCs and M-MDSCs (**D**) in septic (CS 18 h) compared to naive neonatal mice. **F**, **I** Enriched biological pathways (bar graph) in PMN-MDSCs in BCG-vaccinated neonatal mice, prior to sepsis (BCG vs. naive) and after sepsis (BCG + CS18 h vs. CS18 h). **G** Graphical summary of IPA core analysis, showing the major biological entities (p < 0.05) affected by BCG-vaccination compared to naive neonatal mice. Orange predicts activation (z-score ≥ 2.00), blue predicts inhibition (z-score ≤ − 2.00). n = 5–7/group

As previous murine studies have demonstrated that BCG-vaccination induces transcriptional changes in HSCs (Kaufmann et al. 2018), we therefore studied presumed biological functions and processes of splenic myeloid cells based on gene expression patterns. First, we identified the DEGs in PMN- and M- MDSCs among the experimental conditions included in the study (naive, BCG-vaccinated, sepsis, BCG-vaccinated plus sepsis). Comparing unvaccinated septic to healthy neonatal mice, 677 DEGs (525 up- and 152 down-regulated) were observed in PMN-MDSCs (Fig. 7A and C) and 225 DEGs (174 up- and 51 down- regulated) in M-MDSCs (Fig. 7B and D). Overall, the patterns in both MDSC subsets from septic neonatal mice were consistent with a significant pro-inflammatory response (Fig. 7A–D). Significant pathways displaying activation in PMN-MDSCs from septic versus naive neonatal mice included (z-score ≥ 2.00): neutrophil degranulation, IL- 27, IL- 4/IL- 13, S100 family, pathogen-induced cytokine storm, iNOS, glycolysis and IFN alpha/beta (Fig. 7C). Similarly, M-MDSCs from septic neonatal mice showed predictive activation (z-score ≥ 2.00) of: neutrophil degranulation, class I MHC antigen processing and presentation, pathogen induced cytokine storm, T_H2_ and IL- 4/IL- 13 compared to naive healthy neonatal mice (Fig. 7D).

BCG-vaccination in healthy neonatal mice induced 43 DEGs (33 up- and 10 down-regulated) in PMN-MDSCs and 2 DEGs in M-MDSCs in healthy neonatal mice seven days later. These PMN-MDSC DEGs were predicted to be involved in activation of the neutrophil degranulation pathway in PMN-MDSCs (z-score ≥ 2.00; Fig. 7E–G). Following sepsis, BCG-vaccination induced 24 DEGs (7 up- and 17 down-regulated) in PMN-MDSCs and 10 DEGs (1 up- and 9 down-regulated) in M-MDSCs compared to unvaccinated septic mice. BCG-vaccination significantly downregulated expression of genes involved in the neutrophil degranulation pathway in PMN-MDSCs compared to their septic unvaccinated littermates (z-score ≤ − 2.00; Fig. 7H, I).

As BCG induced a significant expansion of myeloid cells and recent studies indicate that BCG-induced trained immunity not only alters the functional program of monocytes but also induces a strong bias to granulopoiesis and reprograms neutrophils (Brook et al. 2020; Kaufmann et al. 2018; Moorlag et al. 2020); we therefore next determined the potential functional implications of BCG-vaccination in neonatal innate immune effector cells. First, we analyzed the effect of sepsis on the functional phenotype of neutrophils, monocytes, macrophages and DCs, which are summarized in the supplementary Table 1. As expected, most of the upregulated pathways (z-score ≥ 2.00) in these cells were those related to a pro-inflammatory response eighteen hours post-sepsis. Whereas BCG-vaccination induced a pro-inflammatory-like phenotype on neutrophils, monocytes, macrophages and DCs, compared to naive neonatal mice (BCG-vaccinated versus naive neonatal mice; supplementary Table 2), BCG-vaccination promoted a shift in the top canonical pathways towards cell-cycle pathways after sepsis. Importantly, this was not demonstrated in macrophages that showed a significant downregulation of degranulation and pathogen-induced cytokine storm signaling pathways (z-score ≤ − 2.00), and an overall predicted downregulation of TLR4 and MyD88 signaling pathways (e.g. migration of granulocytes, recruitment of leukocytes, adhesion and cell movement of neutrophils) (Figure S8 A-B).

Histone modification plays a key role in the epigenetic reprogramming of trained immunity (Ochando et al. 2023). Linker histones of the H1 family (H1) are key components of chromatin that seem to regulate DNA methylation and hence gene expression (Yang et al. 2013). Interestingly, while sepsis-induced upregulation of H1 associated genes in PMN-MDSCs (compared to naive), BCG-vaccination followed by sepsis revealed significantly reduced expression of multiple H1 associated genes (compared to sepsis alone). This was true not only in PMN-MDSCs (e.g. *Hist1 h2ab, Hist1 h2ap, Hist1 h3f, Hist1 h3c*) and M-MDSCs (e.g. *Hist1 h2ap, Hist1 h2ab, Hist1 h2ai, Hist1 h4 d*), but also in all other innate immune effector cells analyzed in this study (supplementary Table 3).

#### Trajectory analysis of scRNAseq data reveals that BCG induces unique myeloid cell differentiation trajectories

To further understand the effect of either BCG-vaccination and/or sepsis on the transcriptional dynamics of individual myeloid cells (based on the ratio of unspliced to spliced mRNAs) we applied RNA trajectory analysis, which predicts the rate and direction of change in gene expression. The trajectory results showed that BCG-vaccination induced both neutrophil and PMN-MDSC differentiation from P1 PMN (most immature granulocytes) prior to sepsis (Figure S9 B). We found that after sepsis induction in naive mice, PMN clusters represent interrelated multiple differentiation stages with a continuous differentiation from P1 PMN (immature) towards P4 PMN (neutrophils) (Grieshaber-Bouyer et al. 2021). While PMN-MDSCs appear to be replenished from PMNs and monocytes in healthy neonatal mice, 18 h after sepsis a lower differentiation activity was found in these cells (Figures S8 A and S8 C). Notably, eighteen hours after sepsis, BCG induced an altered direction of gene expression changes in the myeloid compartment (including MDSCs), which show a trajectory toward neutrophils and PMN-MDSCs. Similarly, we observed directional movement from classical monocytes to non-classical monocytes in the BCG plus sepsis neonatal mice compared to their septic unvaccinated littermates (Figure S9 C-D).

## Discussion

Enhancing the neonatal innate immune response with either nonpathogenic microbes or chemical adjuvants holds promise for preventing sepsis and reducing mortality. In this regard, although BCG-induced trained immunity reduces overall morbidity and mortality caused by infections other than tuberculosis in children (Jensen et al. 2015; Schaltz-Buchholzer et al. 2022; Chen et al. 2023; Kristensen et al. 2000), the molecular mechanisms responsible for this protective effect have not been fully elucidated. As we learn more about the pathophysiology of neonatal sepsis, it is unclear if the increased sepsis occurrence and severity in neonates, especially VLBW, are secondary to a dysregulated inflammatory response that induces a rapid multiorgan injury and mortality, or to a tolerogenic-like immune landscape (required during pregnancy) that is permissive to microbial pathogens or both. Our findings demonstrate that BCG administration significantly protects neonatal mice from sepsis mortality, consistent with randomized controlled trials in Guinea-Bissau, where low-birth-weight (LBW) neonates vaccinated at birth experienced a 40% reduction in mortality (Jensen et al. 2015; Kristensen et al. 2000; Roth et al. 2004). Similar findings have been reported in experimental models of influenza and polymicrobial sepsis (Brook et al. 2020; Kaufmann et al. 2022).

The survival benefit of BCG vaccination is likely multifactorial, involving multiple inflammatory and immune pathways within innate immune effector cells. BCG-induced trained immunity enhances HSPC expansion and myeloid cell activation through pattern recognition receptor (PPR)-mediated signaling, triggering metabolic and epigenetic reprogramming (Riksen and Netea 2021). In neonatal mice, BCG vaccination induces a modest systemic inflammatory response and expansion of multipotential progenitors (LSK), myeloid precursors (GMP), MDSCs (CD11b^+^Gr1^+^MHCII^dim/−^) and differentiated myeloid cells, including PMN neutrophils, monocytes and macrophages. These findings recapitulate what is seen in humans, where BCG vaccination in newborns induces transcriptomic, epigenomic and functional reprogramming of HSPC and peripheral monocytes, hallmarks of trained immunity (Cirovic et al. 2020). More recently, it has been shown that BCG-induced trained immunity and emergency myelopoiesis are interconnected in neonatal and adult sepsis models; however, the precise mechanisms underlying nonspecific protection remain unclear, especially in neonates (Brook et al. 2020; Andualem et al. 2023).

BCG-vaccination significantly altered the neonatal host response to sepsis by attenuating systemic inflammation and reducing splenic inflammatory monocytes (CD11b^+^Ly6 C^hi^), concomitant with the expansion of CD11b^+^Gr1^+^MHC^dim/−^ and downregulation of inflammatory pathways in PMN-MDSCs and macrophages. Given that during neonatal sepsis the major organ where extramedullary myelopoiesis takes place is the spleen, that reaches its peak at six hours post-sepsis, with the current data we are not able to elucidate the impact of sepsis or BCG-induced trained immunity in other cell compartments (e.g. blood, peritoneal cavity).

The effects of the BCG vaccine on myeloid cells are known to occur via stimulation of multiple pathogen recognition receptor (PRRs) and nucleotide-binding oligomerization domain (NOD)-like receptors (NLRs) (Covián et al. 2019). BCG-induced immune activation was characterized by increased production of pro-inflammatory cytokines, including IL- 1β, TNFα, MCP- 1 and IL- 8 (CXCL8) (Bulut et al. 2005). Importantly, following sepsis, BCG-vaccinated neonatal mice showed significantly lower circulating levels of IL- 6, KC and M-CSF compared to unvaccinated septic controls; thus, the protection of BCG in our model of neonatal sepsis may be, in part, via attenuation of the systemic inflammation as reported previously (Koeken et al. 2020). Mechanistically, it has also been suggested that BCG-mediated reductions in inflammation could explain the reduced morbidity associated with SARS-CoV- 2 infection and COVID- 19 severity (Netea et al. 2020; Kleen et al. 2020).

S100 alarmins (especially S100 A8 and S100 A9) are endogenous alarmins specifically linked to both antimicrobial and inflammatory activities (Yang et al. 2017). In healthy neonates, circulating levels of S100 A8/A9 are significantly higher than in adults (Austermann et al. 2014), which induces a transient tolerogenic immune state that protects newborns against hyperinflammatory immune responses by promoting MDSC expansion and function (Ulas et al. 2017; Sinha et al. 2008; Gabrilovich and Nagaraj 2009). In our neonatal sepsis model, BCG vaccination further increased S100 A9 levels both prior to and slightly after sepsis induction, which was associated with the reduction of inflammatory monocytes. In this regard, Heinemann and colleagues recently showed that neonatal S100 A8/S100 A9 alarmins prevented the expansion of inflammatory monocytes, which has been implicated in fatal neonatal sepsis due to systemic hyperinflammatory responses (Heinemann et al. 2017). While neonatal sepsis is associated with increased production of pro-inflammatory and anti-inflammatory cytokines, it is unclear how the imbalance between these two responses is related to sepsis severity (Hibbert et al. 2018). There is evidence that mortality is due to an overwhelming hyper-inflammatory immune response; however, sepsis-induced immunosuppression has also been proposed as a contributing factor (Hibbert et al. 2018).

Substantial evidence suggests that BCG’s protective mechanisms also include expansion and transcriptional reorganization of multiple myeloid cell subsets. BCG vaccination was associated with a marked expansion of both undifferentiated and differentiated myeloid subsets, including splenic CD11b^+^Gr1^+^ cells, PMNs, monocytes and macrophages. While BCG modestly increased PMN- and M- MDSCs in healthy mice, post-sepsis MDSC numbers were essentially unchanged. Transcriptional trajectory analysis revealed a continuous differentiation pathway from immature (P1 PMN) to mature (P4 PMN) neutrophils in both naive and septic neonatal mice. Whereas PMN-MDSCs were replenished from PMNs and monocytes in healthy neonatal mice, in septic BCG-vaccinated neonatal mice, gene expression changes favored neutrophil and PMN-MDSC differentiation.

Transcriptional responses of multiple myeloid subsets showed a strong interactive effect between BCG vaccination and sepsis. Whereas sepsis was associated with the up regulation of genes associated with pathogen-associated cytokine storm, neutrophil degranulation, iNOS, glycolysis and IFNa/b signaling pathways, BCG vaccination shifted the top canonical pathways toward cell-cycle regulation, except in macrophages, where degranulation and pathogen-induced cytokine storm signaling pathways were downregulated. Notably, BCG-vaccinated neonatal mice exhibited reduced TLR4/MyD88 signaling and suppression of pathways linked to granulocyte migration and recruitment, neutrophil adhesion and movement, compared to sepsis alone.

Importantly, CD11b^+^Gr1^+^ splenocytes from both naive and BCG vaccinated mice flipped from being net T-cell immunosuppressive to stimulating T-cell proliferation ex vivo after sepsis. In addition, oxygen consumption and therefore, energy expenditure in CD11b^+^Gr1^+^ splenocytes from BCG vaccinated mice after sepsis were dramatically increased consistent with increased oxygen consumption rates (mitochondrial respiration) and glycolysis. Reactive oxygen and nitrogen species play a central role in antimicrobial activities of myeloid populations. During sepsis (in adults), metabolic reprogramming occurs in immune effector cells that switch to glycolysis as their primary source of energy (the Warburg effect) (Bar-Or et al. 2018); conversely, neonatal immune response is programed to a ‘disease tolerance’ state potentially due their low energy reservoir (Conti et al. 2020). Upregulation of glucose metabolism has been associated with chromatin modifications seen during BCG-induced trained immunity (Kühtreiber et al. 2020; Moorlag et al. 2024). However, how long these effects last after sepsis remains unknown and therefore, future research (including epigenetic modifications in innate immune cells) is warranted.

In contrast to our initial hypothesis that BCG’s survival benefit being only secondary to induction of inflammation and trained immunity, these findings suggest that the host protective effects are more nuanced and widespread. BCG protection was likely mediated by a reduction of the inflammatory burden via not only MDSC expansion and activation (demonstrated by their high rates of both glycolysis and oxidative phosphorylation), but also a net shift from T-cell immune suppression to activation. The overall myeloid cell compartment signature after BCG vaccination showed predicted activation of pro-inflammatory pathways 1 week after BCG-vaccination; nevertheless, eighteen hours following sepsis BCG significantly reduces inflammation and accelerates protein synthesis and cell cycle activity. BCG also induces expansion of HSCs and drives differentiation of immature PMNs and expansion of nontraditional monocytes. The cellular and molecular mechanisms responsible for the beneficial effects of BCG against secondary infections include transcriptional, epigenetic (methylation, acetylation) and metabolic reprogramming of myeloid cells. Our data show that BCG induces downregulation of multiple histones of the linker H1 family genes in myeloid cells (including MDSCs), which are associated with the G1 arrest on these cells. H1 histones are critical regulators of gene silencing via control of chromatin architecture (methylation/acetylation) and epigenetic landscape, which orchestrate most chromatin-related cellular events (Willcockson et al. 2021). Since histone modifications are key epigenetic mechanisms involved in trained immunity, these findings warrant further research.

Our study has several limitations. First, we did not quantify bacterial loads but used other biomarkers to assess sepsis severity in our murine model of neonatal sepsis. As frequently seen in newborns (especially in preterms), the lack of consensus for both neonatal sepsis definition and the sensitivity of blood cultures is suboptimal; therefore, clinical suspicion frequently leads to empiric antibacterial therapy, even with negative cultures (often referred to as “culture-negative sepsis”) (Molloy et al. 2020). In our work, circulating cytokine production significantly increased in septic neonatal mice as early as six hours post-CS, which correlated with initiation of a prodromal syndrome (scattering of the pup or the absence of a milk spot) leading to abandonment and the requirement for euthanasia. Second, we used Gr1 and CD11b as surface markers for isolating MDSCs for ex-vivo functional analysis. This has limitations as these two markers don’t fully capture the heterogeneity and complexity of neonatal murine MDSCs, as we showed using scRNA-seq technology. Our novel neonatal MDSCs in the context of sepsis warrants further investigation into the role of myeloid cells (including MDSCs) not only earlier after sepsis induction, but when the resolution of the inflammatory insult is reached.

In conclusion, this study provides neonatal myeloid cell compartment (including MDSCs) transcriptional landscape and function in a murine model of neonatal polymicrobial sepsis. Although neonatal MDSCs’ research has gradually increased over the last 10 years, the role of these cells at birth is still largely unknown. Employing BCG-induced trained immunity we show the systemic nature by which BCG impacts the neonatal immune response to sepsis and induces an immunosuppressive phenotype in myeloid cells, which might be a mechanism responsible (in part) for the survival benefit to sepsis seen in neonatal mice vaccinated with BCG at birth. The impact of trained immunity on myeloid cells is complex due to the dynamic nature of continuous cells differentiation in neonatal spleens; therefore, future work considering the study of gene landscape in progenitor cells are needed to understand cell reprogramming during trained immunity, which might provide relevant understanding for therapeutic interventions to prevent sepsis in neonates.

## Supplementary Information

Supplementary material 1.

Supplementary material 2.

Supplementary material 3.

## Data Availability

The raw sequencing data in the form of FASTQ files, have been deposited in NCBI’s Gene Expression Omnibus (GEO) and are accessible through GEO Accession Number GSE276868.
