# Supplementary material for: Innate immune training in the neonatal response to sepsis
Source: Mol Med. 2025 Apr 30;31:159. doi: 10.1186/s10020-025-01179-5 (PMC12042443; doi:10.1186/s10020-025-01179-5)
Supplement: Supplementary file 1 — Supplementary material 1. [file 10020_2025_1179_MOESM1_ESM.docx]

**Supplementary Key Resources Table**

1. Antibodies (dilution 1/100)

| **Target** | **Clone** | **Fluorophore** | **Identifier** |
| --- | --- | --- | --- |
| Mouse CD3e | 145-2C11 | n/a | BD Biosciences; 553057 |
| Mouse CD28 | 37.51 | n/a | BD Biosciences; 553294 |
| Mouse CD4 | RM4-5 | APC | BD Biosciences; 553051 |
| Mouse CD8a | 53-6.7 | PE | BD Biosciences; 553032 |
| Mouse I-A/I-E | M5/114.15.2 | Alexa Fluor® 700 | Biolegend; 107622 |
| Mouse F4/80 | BM8 | PE | Biolegend; 123110 |
| Mouse CD11c | HL3 | Bv421 | BD Biosciences; 562782 |
| Mouse CD11b | M1/70 | PE-Cy7 | BD Biosciences; 552850 |
| Mouse Ly6G | 1A8 | APC | BD Biosciences; 560599 |
| Mouse Ly6C | AL-21 | FITC | BD Biosciences; 553104 |
| Mouse CD86 | GL-1 | PE | Biolegend; 105007 |
| Mouse CD80 | 16-10A1 | FITC | BD Biosciences; 561954 |
| Mouse Lin-1 | 145-2C11; RB6-8C5; RA3-6B2; Ter-119; M1/70 | FITC | Biolegend; 133301 |
| Mouse CD117 (c-kit) | 2B8 | PerCP | Biolegend; 105821 |
| Mouse CD34 | HM34 | PE | Biolegend; 128609 |
| Mouse CD135 | A2F10 | Bv421 | Biolegend; 135313 |
| Mouse CD127(IL-7Rα) | A7R34 | APC | Biolegend; 135011 |

2. Other reagents

| **Reagent** | **Identifier** |
| --- | --- |
| Live-attenuated BCG vaccine, TICE® strain | Merck #NDC 0052-0603-01/02 |
| Mouse Luminex® Discovery Assay – Mouse premixed multi-analyte kit | R&D systems; LXSAMSM |
| EasySep^TM^ mouse T cell isolation kit | STEMCELL Technologies #19851 |
| EasySep^TM^ mouse MDSC (CD11b^+^Gr1^+^) isolation kit | STEMCELL Technologies #19867 |
