# Supplementary material for: Innate immune training in the neonatal response to sepsis
Source: Mol Med. 2025 Apr 30;31:159. doi: 10.1186/s10020-025-01179-5 (PMC12042443; doi:10.1186/s10020-025-01179-5)
Supplement: Supplementary file 2 — Supplementary material 2. [file 10020_2025_1179_MOESM2_ESM.docx]

**Supplementary table 1.** Effect of polymicrobial sepsis (CS18h vs. naive) on the transcriptomic profile of neonatal splenic innate immune effector cells

| **Cell type** | **# DEGs** | **Top canonical pathways** | **z-score** | **p-value** |
| --- | --- | --- | --- | --- |
| Neutrophils | 583 (495 up & 88 down) | *Upregulated:*  - Neutrophil degranulation  - IL-10 signaling  - IFN α/β signaling  - Pathogen induced cytokine storm  - IL-4/IL-13 signaling  - Interferon signaling  - iNOS signaling  - Toll-like receptor signaling  - Acute phase response signaling  - IL-6 signaling  *Downregulated:*  - PPAR signaling | 7.070  3.606  4.690  5.864  4.264  3.051  3.464  2.530  2.683  2.500  -2.496 | 4.21E-31  9.25E-25  3.28E-19  2.41E-17  8.66E-15  2.71E-14  9.83E-14  2.24E-12  6.66E-11  2.13E-10  1.92E-07 |
|  |  |  |  |  |
| Classical Monocytes | 852 (603 up & 249 down) | *Upregulated:*  - iNOS signaling  - IL-10 signaling  - IL-4/IL-13 signaling  - Glycolysis I  - Toll-like receptor signaling  - S100 family signaling pathway  - Glucose metabolism  - Regulation of TLR by endogenous ligand  - MyD88:MAL (TIRAP) cascade initiated on plasma membrane  - MIF regulation of innate immunity  *Downregulated:*  - DAG and IP3  - Effects of PIP2 hydrolysis  - GP6 | 2.530  2.449  3.500  2.828  2.333  2.143  2.309  2.449  2.121  2.828  -2.000  -2.000  -2.121 | 2.59E-07  3.33E-03  6.01E-07  1.64E-06  8.43E-06  8.90E-06  1.10E-05  4.32E-05  5.64E-05  7.99E-05  1.05E-02  1.10E-02  2.41E-02 |
|  |  |  |  |  |
| Non-classical  Monocytes | 335 (246 up & 89 down) | *Upregulated:*  - Th2  - Th1  - Pathogen induced cytokine storm  - MHC class II antigen presentation  - EGF signaling  - Antimicrobial peptides  - IL-4 signaling  - MIF regulation of innate immunity  *Downregulated:*  - IL-12 signaling and production in macrophages  - PD-1, PD-L1 cancer immunotherapy pathway | 3.207  2.887  3.300  3.000  2.236  2.000  2.183  2.000  -2.000  -3.162 | 3.58E-09  7.29E-08  2.20E-06  4.30E-05  8.19E-04  9.76E-04  1.67E-03  2.58E-03  9.32E-08  1.45E-06 |
|  |  |  |  |  |
| Macrophages | 816 (552 up & 264 down) | *Upregulated:*  - IL-4 and IL-13 signaling  - Macrophage alternative activation signaling pathway  - Pathogen induced cytokine storm  - S100 family signaling  - IL-6 signaling  - IL-33 signaling  - Phagosome formation  - IL-10 signaling  - Acute phase response signaling  - STAT3 pathway  *Downregulated:*  - PPAR signaling  - Inhibition of matrix metalloproteases  - PTEN signaling | 2.502  2.058  4.025  4.589  3.578  3.138  3.101  3.051  4.146  2.309  -3.207  -2.449  -2.333 | 6.18E-17  2.00E-16  1.17E-14  7.66E-13  1.10E-10  1.96E-10  5.11E-10  8.16E-10  5.01E-09  2.71E-07  7.09E-06  1.95E-04  3.22E-04 |
|  |  |  |  |  |
| DC | 271 (242 up & 29 down) | *Upregulated:*  - STAT3 pathway  - Interferon alpha/beta signaling  - IL-27 signaling pathway  - Interferon signaling  - iNOS signaling  - Interferon gamma signaling  - Pathogen induced cytokine storm signaling pathway  - Acute phase response signaling  -Th1 pathway  -IL-6 signaling | 2.333  2.530  2.887  2.646  2.449  2.333  3.873  2.333  2.121  2.121 | 3.00E-09  5.06E-09  1.67E-08  1.07E-07  7.24E-07  8.87E-07  1.52E-05  3.74E-05  5.85E-05  1.23E-04 |
|  |  |  |  |  |
| pDC | 193 (88 up & 105 down) | *Upregulated:*  - IL-27 signaling pathway  - Interferon alpha/beta signaling  - Interferon gamma signaling  - Senescence-Associated secretory phenotype  *Downregulated:*  - RHO GTPase cycle | 2.530  2.646  2.236  2.000  -2.121 | 5.63E-08  1.21E-06  7.88E-04  2.40E-03  2.30E-02 |

**Supplementary table 2.** Effect of BCG vaccination on the transcriptomic profile of neonatal splenic innate immune effector cells prior to sepsis (BCG vs. naive) and after sepsis (BCG+CS18h vs. CS18h).

| **Cell type** | **DEGs #** | **Top canonical pathways** | | **DEGs #** |
| --- | --- | --- | --- | --- |
|  |  | **BCG vs Naive** | **BCG+CS18h vs. CS18h** |  |
| Neutrophils | 74  (34 down & 40 up) | - IL-4/IL-13 signaling  - STAT3  - Neutrophil degranulation  - Th2 pathway  - Pathogen induced cytokine storm signaling pathway  - Inflammasome pathway  - Th1 and Th2 activation pathway | - Eukaryotic translation termination  - Eukaryotic translation elongation  - Response of EIF2AK4 (GCN2) to aminoacid deficiency  - Selenoamino acid metabolism  - SRP-dependent cotranslational protein targeting to membrane  - Interferon gamma signaling | 34  (23 down & 11 up) |
|  |  |  |  |  |
| Classical Monocytes | 16  (5 down & 11 up) | - Nitric oxide signaling  - Signaling by TGF-beta receptor complex  - Antimicrobial peptides  - Integrin cell surface interactions | - Processing of capped intron-containing pre-mRNA  - Pyrimidine deoxyribonucleotides de novo biosynthesis I  - mRNA capping  - Nucleotide excision repair pathway  - Pyrimidine ribonucleotides interconversion | 23  (17 down & 6 up) |
| Non-classical monocytes | 6  (1 down & 5 up) | - Antimicrobial peptides (z ≥ 2.00)  - Regulation of TLR by endogenous ligand  - RHO GTPases activate NADPH oxidases  - MyD88:MAL (TIRAP) cascade initiated on plasma membrane  - Class I MHC mediated antigen processing and presentation  - S100 family signaling pathway | - None | 8  (8 down & 0 up) |
|  |  |  |  |  |
| Macrophages | 32  (2 down & 30 up) | - Neutrophil degranulation (z ≥ 2.00)  - Antimicrobial peptide (z ≥ 2.00)  - RHO GTPases activate NADPH oxidases (z ≥ 2.00)  - Leukocyte extravasation signaling (z ≥ 2.00)  - S100 family signaling pathway (z ≥ 2.00)  - Class I MHC mediated antigen processing (z ≥ 2.00) | - Neutrophil degranulation (z ≤ -2.00)  - Granulocyte adhesion and diapedesis  - Agranulocyte adhesion and diapedesis  - Regulation of TLR by endogenous ligand  - Antimicrobial peptides  - MyD88:MAL(TIRAP) cascade initiated on plasma membrane  - Pathogen induced cytokine storm signaling pathway (z ≤ -2.00) | 51  (45 down & 6 up) |
| pDC | 6  (0 down & 6 up) | - Antimicrobial peptides  - Regulation of TLR by endogenous ligand  - RHO GTPases activate NADPH oxidases  - MyD88:MAL(TIRAP) cascade initiated on plasma membrane  - Class I MHC mediated antigen processing  - S100 family signaling pathway  - IL-4 and IL-13 signaling pathway  - IL-17 signaling | - mRNA capping  - Nucleotide excision repair pathway  - RNA polymerase III transcription  - RNA polymerase I transcription  - Assembly of RNA polymerase II complex | 9  (8 down & 1 up) |

**Supplementary table 3.** Effect of BCG on histone gene expression in splenic myeloid cells. Differentially expressed (downregulated) histone associated genes in BCG plus sepsis (BCG+CS18h) versus septic (CS18h) neonatal mice.

| **Cell type** | **Gene ID** |
| --- | --- |
| PMN-MDSCs | *Hist1h2ab, Hist1h2ap, Hist1h3f, Hist1h3c, Hist1h1c, Hist1h3e, Hist1h2ae, Hist1h2bg, Hist1h2bb, Hist1h2be, Hist1h4c, Hist1h2ak, Hist1h2bh, Hist1h2ai* |
|  |  |
| M-MDSCs | *Hist1h2ap, Hist1h2ab, Hist1h2ai, Hist1h4d, Hist1h3c, Hist1h3e, Hist1h2ac, Hist1h3f, Hist2h2aa1, Hist1h2bg, Hist1h2ae, Hist1h1c, Hist1h2bn, Hist2h4* |
|  |  |
| Neutrophils | *Hist1h2ap, Hist1h3f, Hist1h3c* |
|  |  |
| Classical monocytes | *Hist1h2ap, Hist1h3c, Hist1h2ae, Hist1h2ab, Hist1h4d, Hist1h2ai, Hist1h3f, Hist1h3e, Hist2h2ac, Hist1h2bg, Hist2h2aa1, Hist1h2an, Hist1h4c, Hist1h2ag, Hist1h4h, Hist1h2bn, Hist1h3a, Hist1h2ah, Hist1h2bm, Hist1h2bj, Hist1h2ac, Hist1h2ak, Hist1h2bb, Hist1h3b, Hist1h4k, Hist1h2bk, Hist2h4, Hist1h3g, Hist2h3b, Hist1h2be, Hist1h4j, Hist1h2bh, Hist1h2af, Hist3h2ba, Hist1h4a, Hist1h4m, Hist1h3i, Hist1h4b, Hist1h4n* |
|  |  |
| Non-classical monocytes | *Hist1h2ab, Hist1h3f, Hist1h2ap, Hist1h4h* |
|  |  |
| Macrophages | *Hist1h2ab, Hist1h3f, Hist1h3e, Hist1h4d, Hist1h3c, Hist1h2ap, Hist2h2ac, Hist1h4c, Hist1h2be, Hist1h2ae, Hist1h2bb, Hist1h2ai, Hist1h4h, Hist1h2bg, Hist1h2bn, Hist1h2bj, Hist1h2ak, Hist1h2bm, Hist2h2aa1* |
|  |  |
| DC | *Hist1h4d, Hist1h3c, Hist1h4h* |
|  |  |
| pDC | *Hist1h2ab, Hist1h3f, Hist1h2ap, Hist1h3e, Hist1h3c, Hist1h4d, Hist2h2ac, Hist2h2ae, Hist1h4c, Hist1h2ai, Hist1h4h, Hist1h4h, Hist1h2bb, Hist1h1c* |
