# Supplementary material for: Innate immune training in the neonatal response to sepsis
Source: Mol Med. 2025 Apr 30;31:159. doi: 10.1186/s10020-025-01179-5 (PMC12042443; doi:10.1186/s10020-025-01179-5)

**SUPPLEMENTARY FIGURE LEGENDS**

**Supplementary Figure 1.** Effect of the heat-inactivated BCG (HI-BCG) in survival following intrabdominal poly-microbial sepsis in neonatal mice. We analyzed the effect of the heat-inactivated BCG administration either (A) at birth (P1) or (B) at day of life 4 (P4) in sepsis mortality induced by cecal slurry (CS) on day of life 7 (P7). Kaplan-Meier survival curves estimated over the lifespan after sepsis induction. Survival data were compared via the Log-rank test. ns: not significant.

**Supplementary Figure 2.** Effect of polymicrobial neonatal sepsis induced by cecal slurry (CS) in circulating cytokine/chemokine concentrations. Seven days old neonatal mice received 1.1-1.3 mg/g BW of CS to induce sepsis. Blood was collected prior to- (Naive), six (CS 6h) and eighteen hours (CS 18h) post-sepsis induction and changes in concentration of circulating cytokines (A-H), growth factors (I-J) and chemokines (K) were determined using a customized mouse Luminex® discovery assay. n=4-7 per group per time point. Error bars represent median and interquartile range. ***p<0.001; **p<0.01; *p<0.05; ##p<0.01 vs. Naive.

**Supplementary Figure 3.** Effect of sepsis and BCG in splenic myeloid cell numbers. Neonatal mice received either BCG or saline at birth and 7 days later were challenged with intra-abdominal polymicrobial sepsis (CS). A. Splenocytes were collected prior to- (Naive or BCG-vaccinated [BCG]), six hours (CS 6h) and 18 hours (CS 18h) post-sepsis induction and analyzed by multicolor flow cytometry for expression of CD11b, Ly6G, Ly6C and F4/80. B. (i) Neutrophils (Ly6G^+^CD11b^+^), (ii) monocytes (Ly6C^+^CD11b^+^), (iii) macrophages (F4/80^+^CD11b^+^) and (iv) inflammatory monocytes (Ly6C^hi^CD11b^+^) were quantified using flow cytometry. n=6-7 per group per time point. Error bars indicate SEM. ***p<0.001; **p<0.01; *p<0.05. ##p<0.01 and #p<0.05 vs. unvaccinated neonatal mice.

**Supplementary Figure 4.** A) Phenotypic characterization of murine splenic hematopoietic stem and progenitor cells (HMPCs) in the spleen. Murine neonatal splenocytes collected prior to- (Naïve or BCG-vaccinated [BCG]) and 18 hours after sepsis were stained with fluorochrome-conjugated antibodies to evaluate the expression of c-kit on lineage and Sca-1 double negative cells. GMP and CMP cells were identified by the expression of CD34 and CD16/32 surface markers. B) BCG-vaccination induces expansion of splenic lineage^-^sca-1^+^c-kit^+^ (LSKs) in neonatal mice. Neonatal mice received either BCG or saline at birth and seven days later were challenged with intra-abdominal polymicrobial sepsis (cecal slurry; CS). Spleens were collected prior to- and 18 hours post-sepsis. Splenic (i) LSKs, (ii) CMPs and (iii) GMPs were quantified using flow cytometry. Error bars indicate SEM. **p<0.01; *p<0.05; ##p<0.01 vs. Naïve; #p<0.05 vs. Naive.

**Supplementary Figure 5.** Splenic MDSCs identification using transcriptomic analysis in young adult mice (as reference). A) Sepsis was induced in adult mice using the CLP model followed by daily chronic stress for seven days (DCS7d), when spleens from naïve and septic young adult mice were harvested and processed using 10X Genomics chemistry (n=8-10/group). B) UMAP visualization showing M- and PMN- MDSCs that are significantly over-represented in adult septic mice (ii) compared to naïve (i). C) Bars with error bars represent the mean ± SEM values for M-MDSCs and (D) PMN-MDSCs in naïve and septic (CLP+DCS7d) adult mice. ****p<0.0001. n=8-10/group. CLP: cecal ligation and puncture; DCS7d: daily chronic stress for seven days; scRNAseq: single-cell RNA sequencing.

**Supplementary Figure 6.** A) UMAP visualization showing M- and PMN- MDSCs that are significantly over-represented in neonatal compared to adult naïve mice. B-C) Quantitative bar plots show the comparison of proportions of splenic M-MDSCs and PMN-MDSCs using transcriptomics, in neonatal (Naive, CS 18h, BCG, BCG+CS 18h) and naïve young adult mice. ***p<0.001 and **p<0.01 vs. Naïve neonatal mice. Bars with error bars represent the mean ± SEM values. n=5-7/group for neonatal mice. n=8 for adult naïve mice.

**Supplementary Figure 7.** Proportion of monocytes and PMNs in murine neonatal spleen using transcriptomics. Bar plots showing the proportion of monocytes (A) and PMNs (C) clusters. The bar plots (B and D) show the ratio of classical monocytes and neutrophils (P4 PMN), respectively. Bars with error bars represent the mean ± SEM values. ***p<0.001; **p<0.01; p<0.05. n=5-7/group.

**Supplementary Figure 8.** A) BCG followed by sepsis induced a significant downregulation of neutrophil degranulation and pathogen-induced cytokine storm signaling pathways (z-score < - 2.00) in macrophages and an overall predicted downregulation of the major biological pathways in our IPA core analysis. n=5-7/group.

**Supplementary Figure 9.** Trajectory analysis reveals the shifting of myeloid cell phenotypes in sepsis and the effect of BCG. RNA velocity analysis for myeloid cells is shown in (A) naïve, (B) BCG-vaccinated, (C) septic (CS 18h) and (D) BCG plus sepsis (BCG + CS 18h) neonatal mice, based on Monocle2.

**SUPPLEMENTARY FIGURE 1**


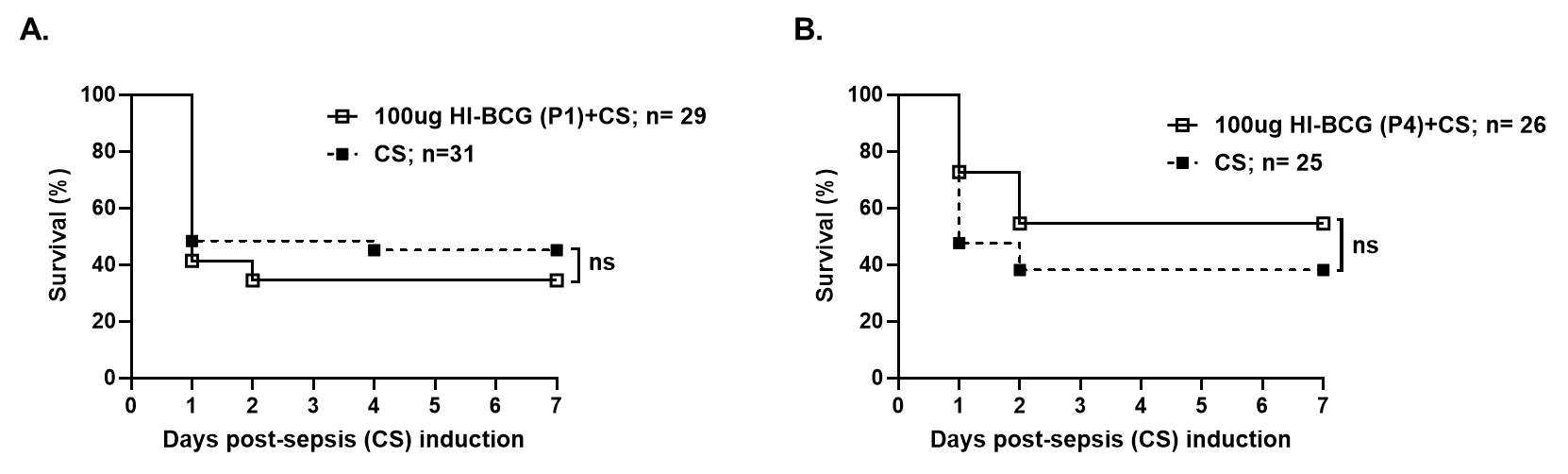


**SUPPLEMENTARY FIGURE 2**

**
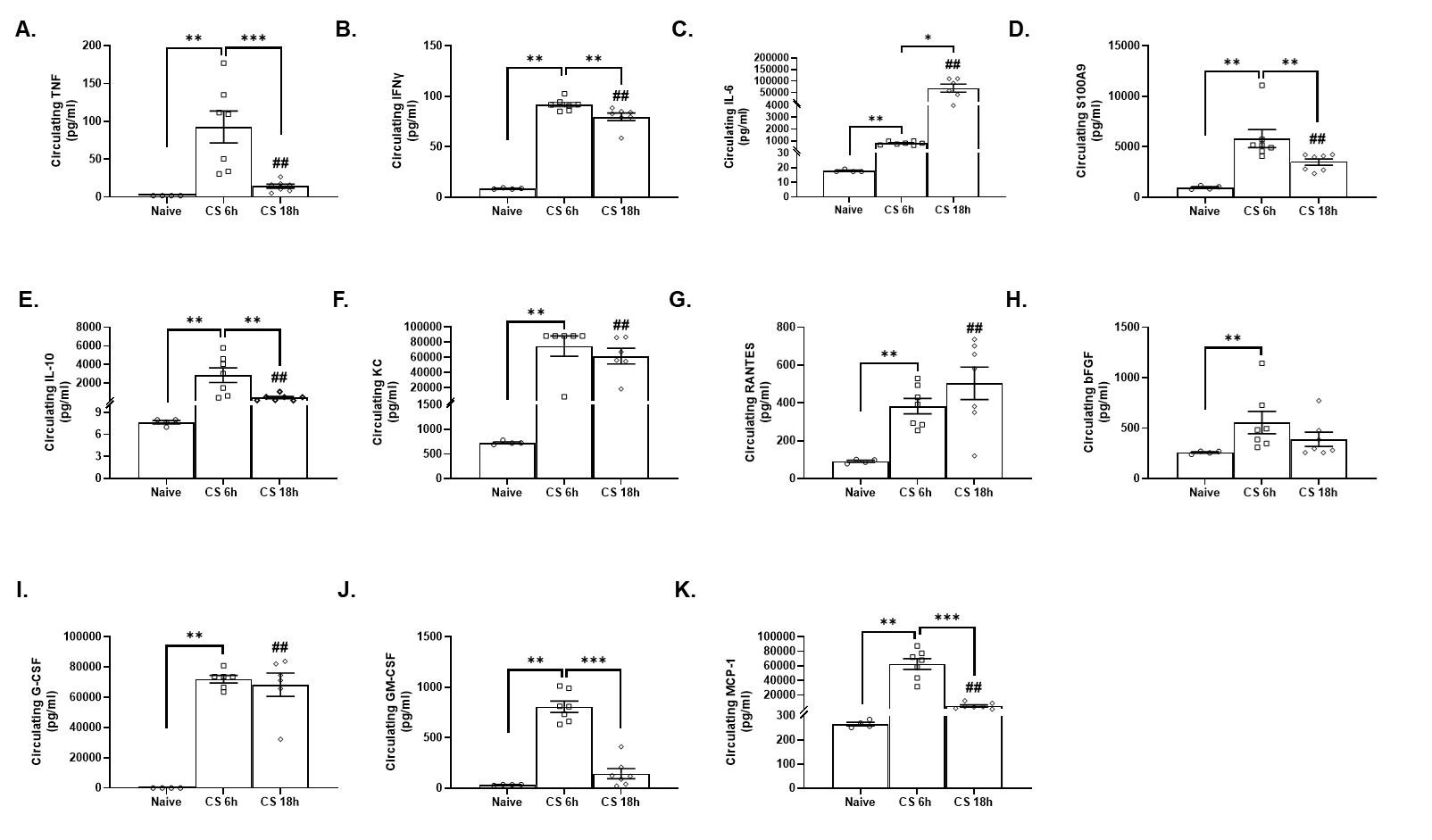
**

**SUPPLEMENTARY FIGURE 3**

**A.**

**
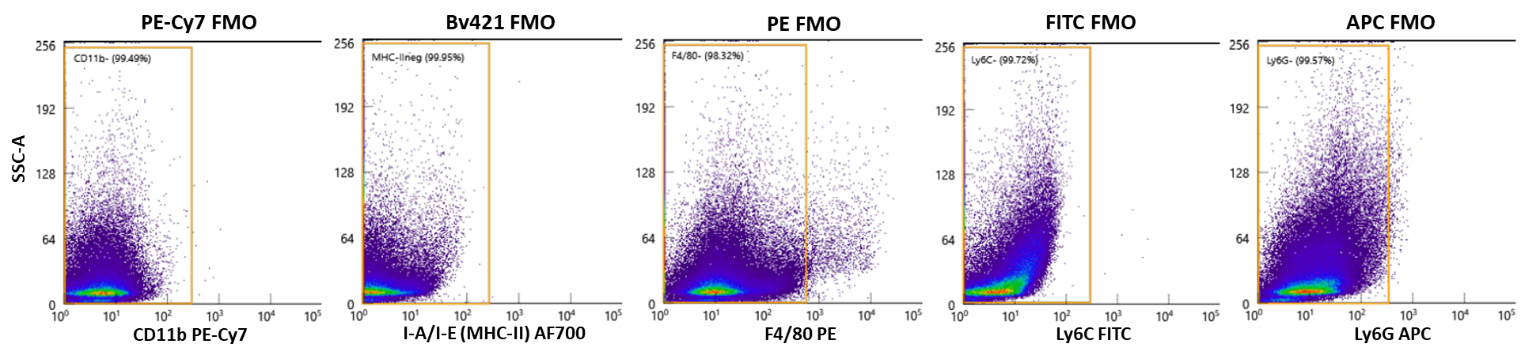
**

**B.**

**
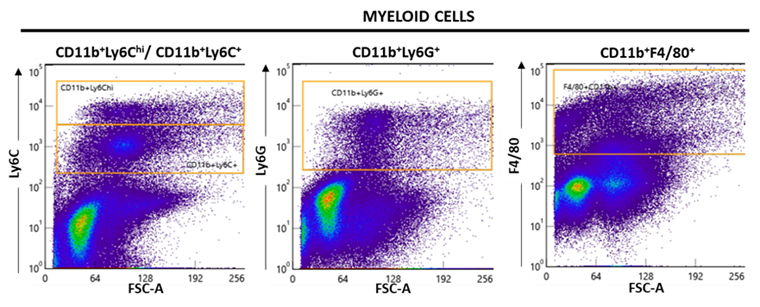
**

**C.**

**
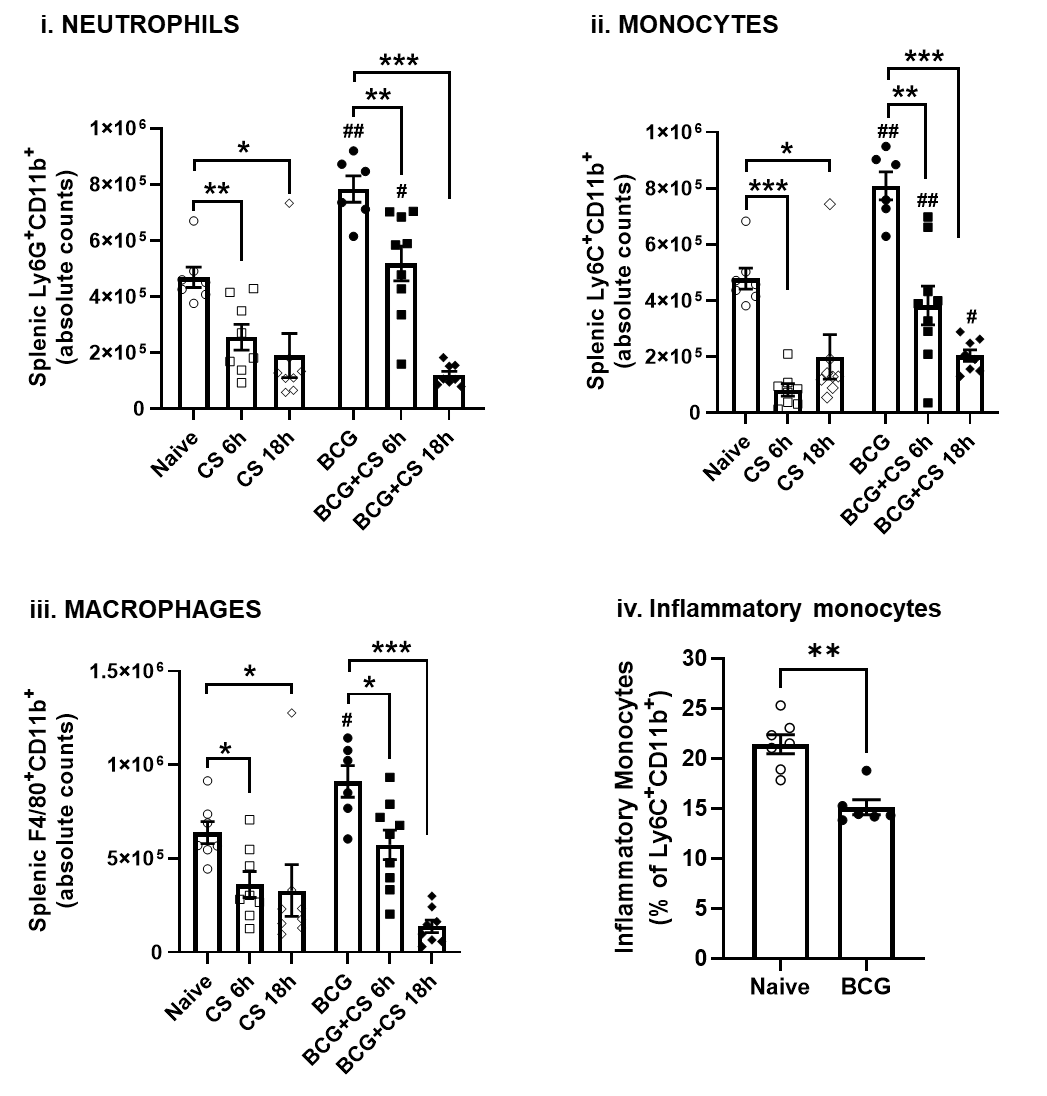
**

**SUPPLEMENTARY FIGURE 4**

**A.**

**
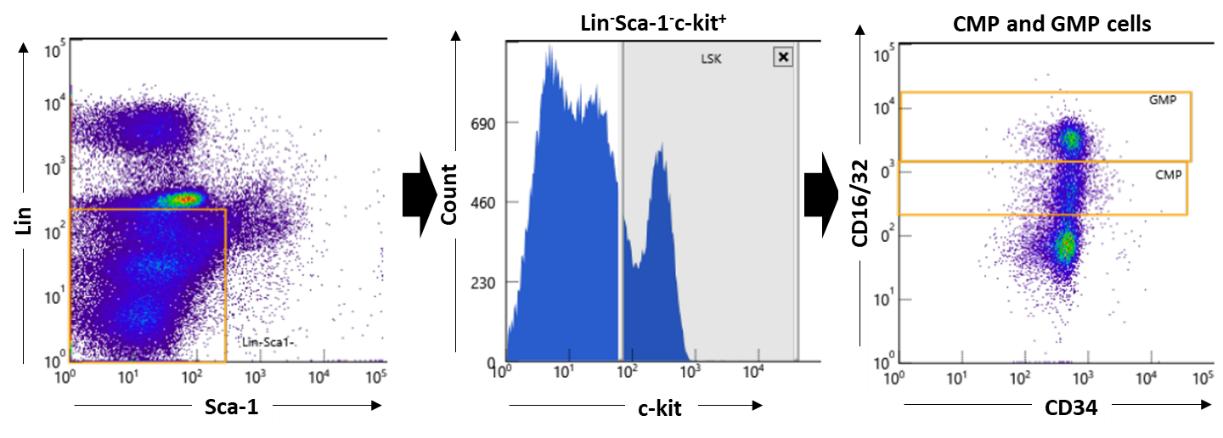
**

**B.**

**
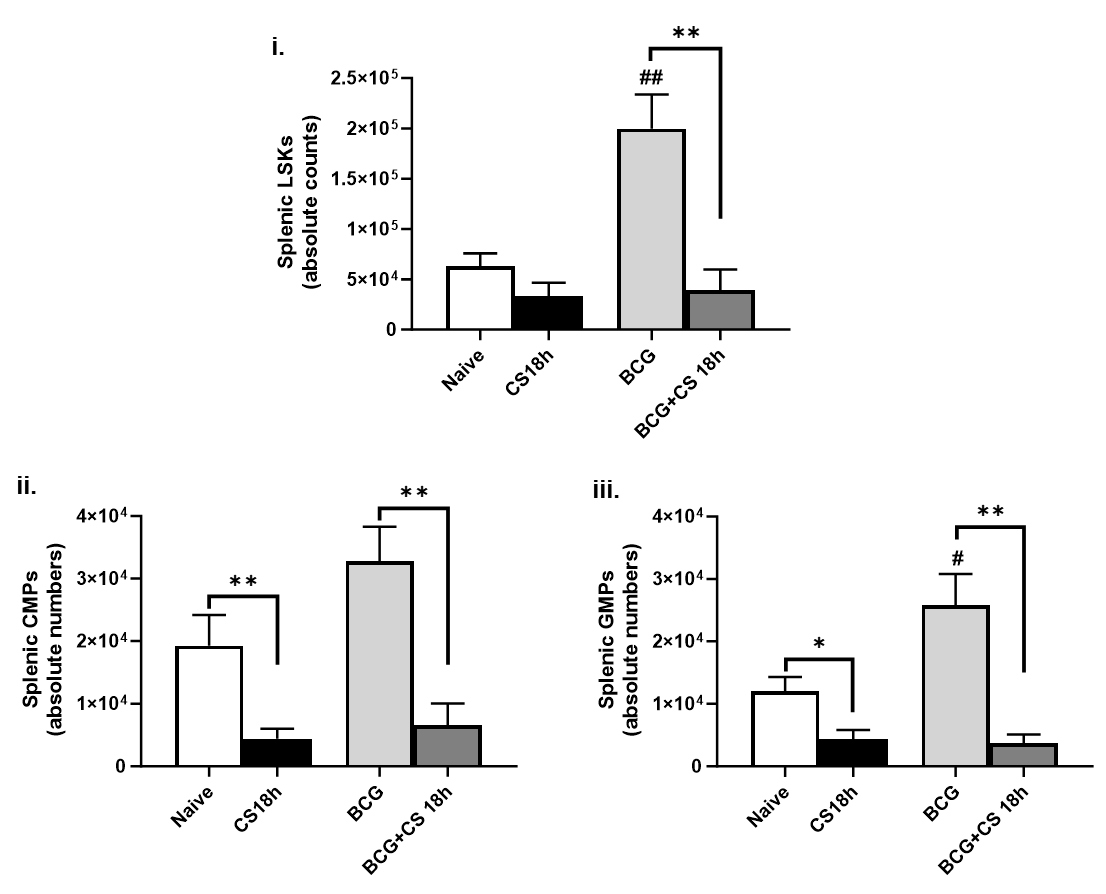
**

**SUPPLEMENTARY FIGURE 5**


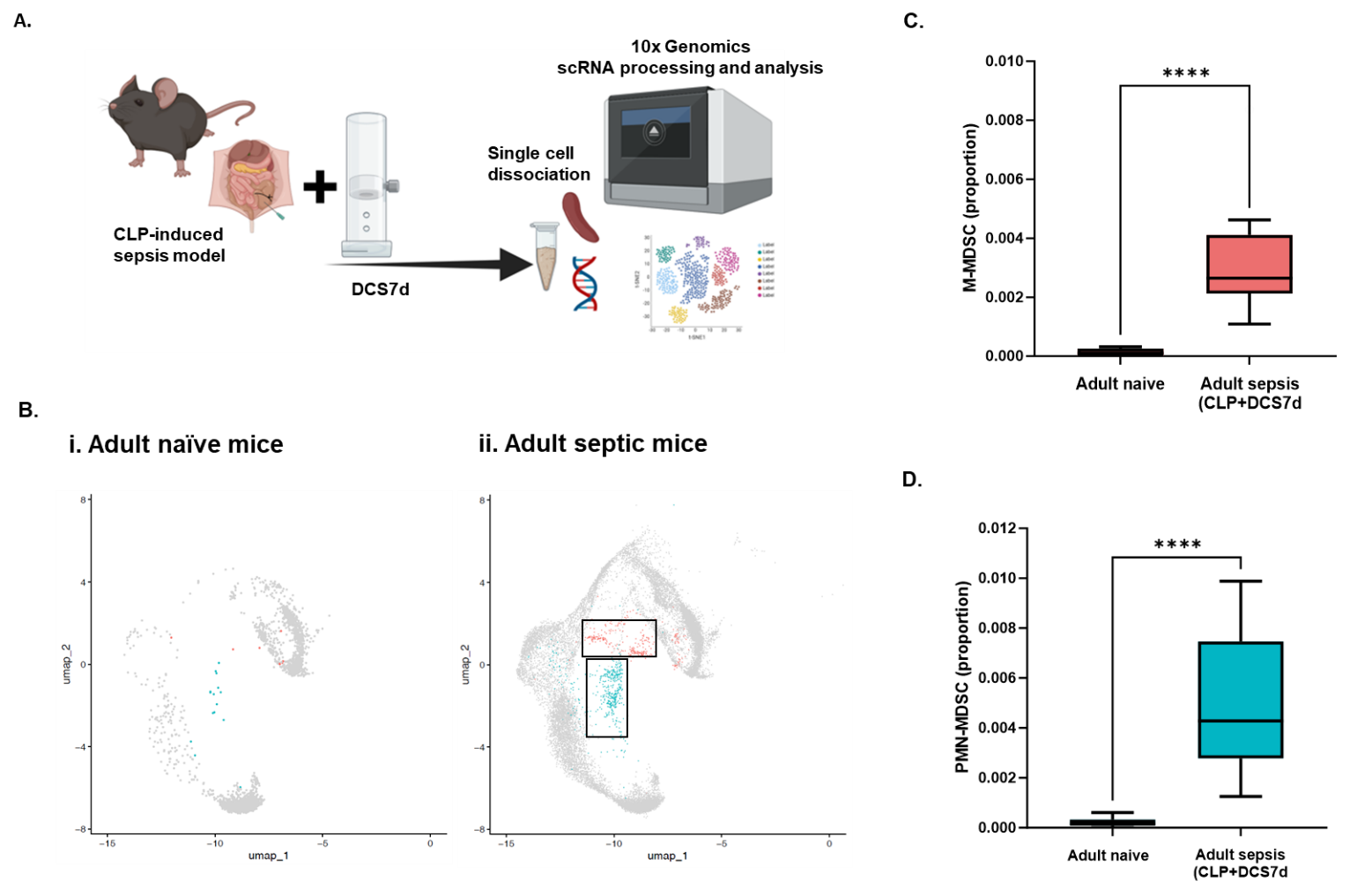


**SUPPLEMENTARY FIGURE 6**


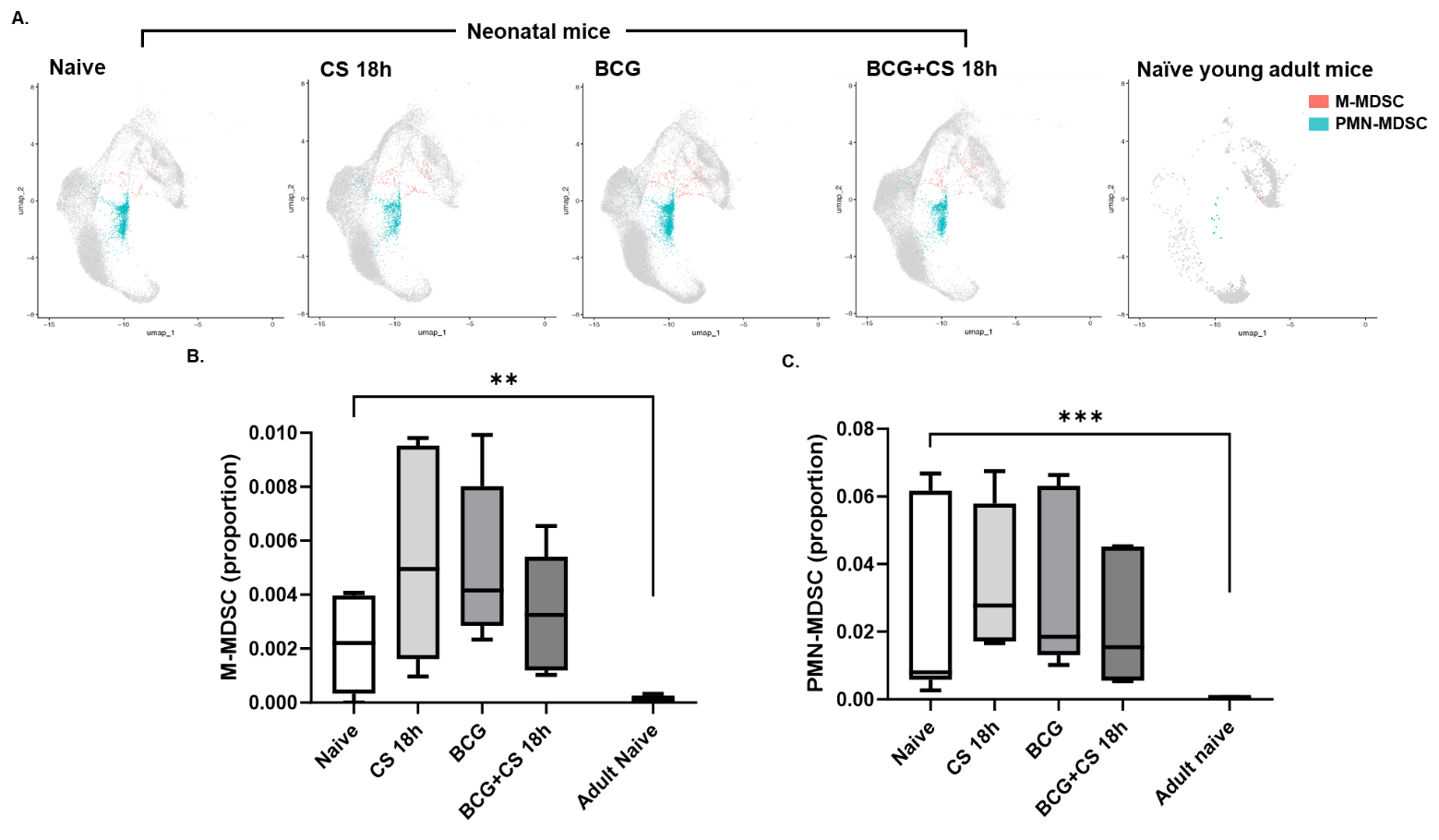


**SUPPLEMENTARY FIGURE 7**


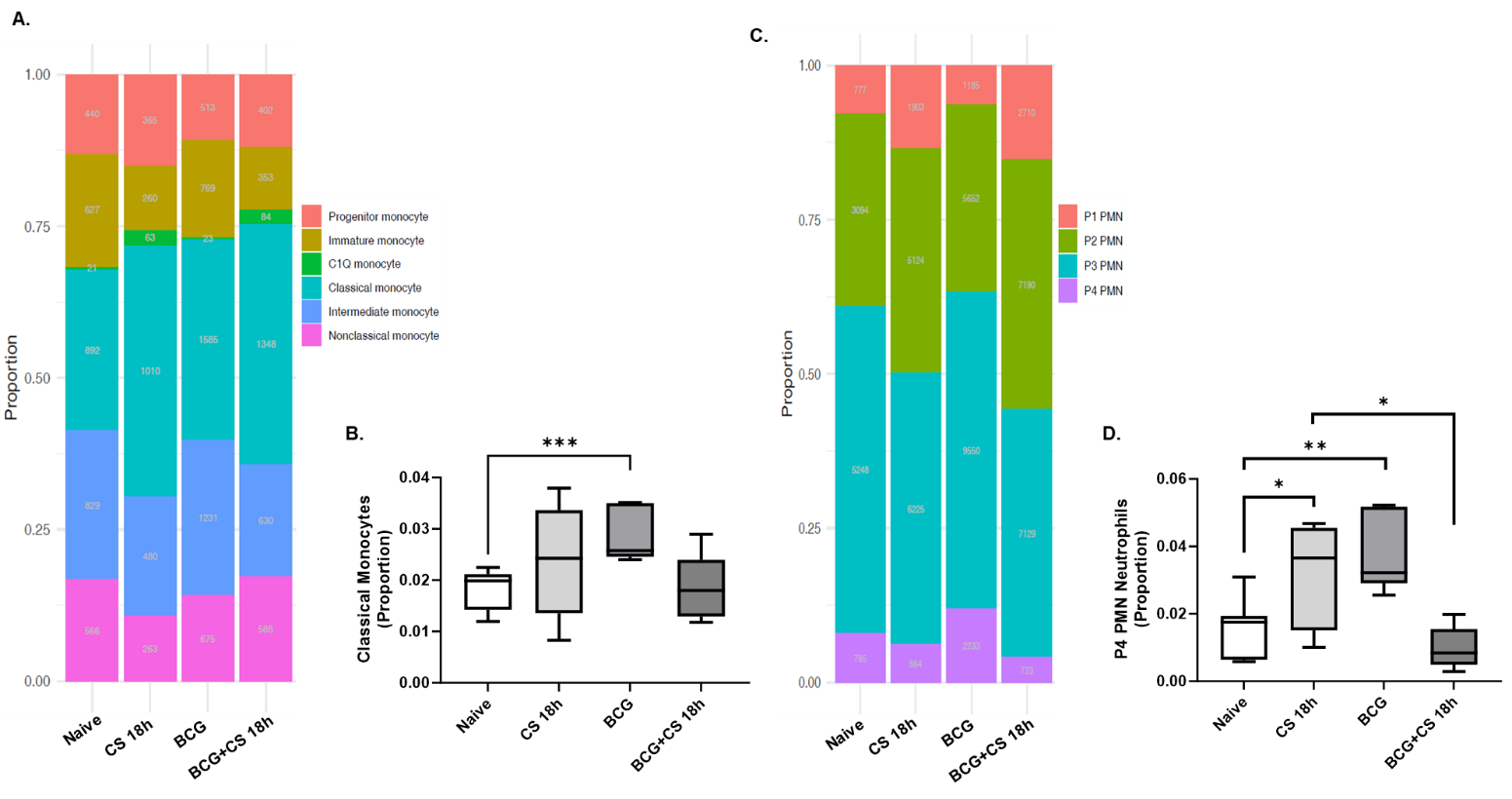


**SUPPLEMENTARY FIGURE 8**

**
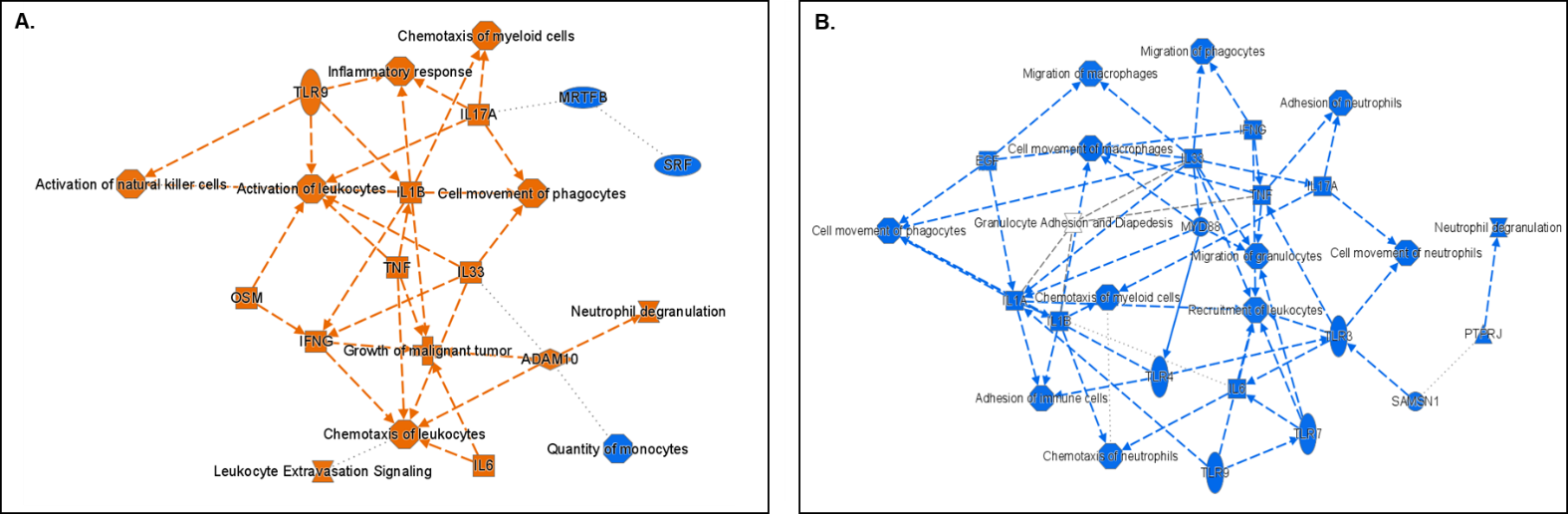
**

**SUPPLEMENTARY FIGURE 9**
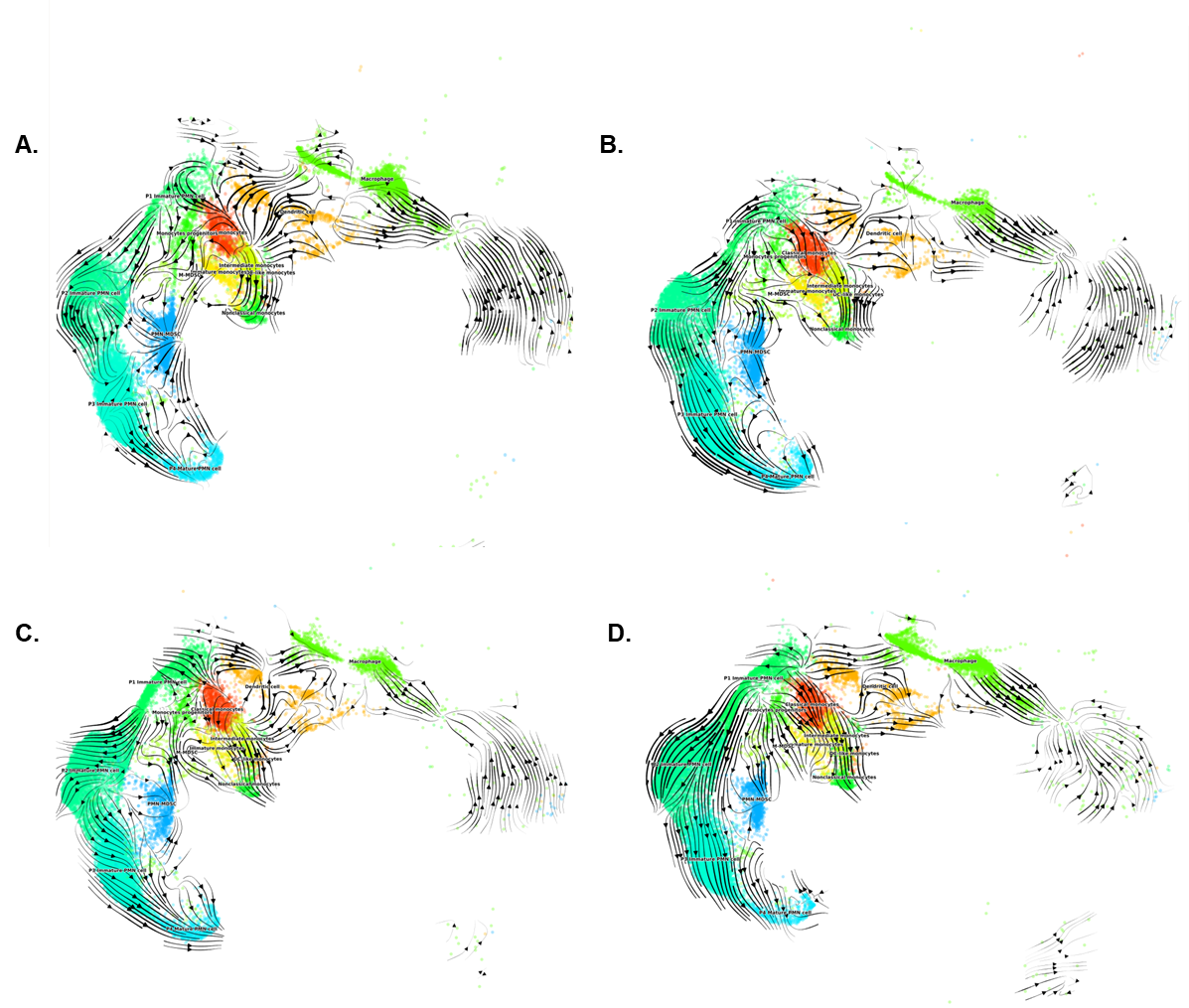

Supplement: Supplementary file 3 — Supplementary material 3. [file 10020_2025_1179_MOESM3_ESM.docx]
